# Prevalence and antibiotic resistance of *Staphylococcus aureus* associated with a college-aged cohort: life-style factors that contribute to nasal carriage

**DOI:** 10.3389/fcimb.2023.1195758

**Published:** 2023-06-27

**Authors:** Sean T. Congdon, John A. Guaglione, Omario M. A. Ricketts, Kyle V. Murphy, Megan G. Anderson, Darby A. Trowbridge, Yousuf Al-Abduladheem, Annabelle M. Phillips, Allison M. Beausoleil, Alexus J. Stanley, Timothy J. Becker, Adam C. Silver

**Affiliations:** ^1^Department of Biology, University of Hartford, West Hartford, CT, United States; ^2^Department of Computing Sciences, University of Hartford, West Hartford, CT, United States

**Keywords:** *Staphylococcus aureus*, nasal carriage *Stapylococcus aureus*., antibiotic resistance, life-style factors, prevalence, methicillin-resistant *Staphylococcus aureus* (MRSA)

## Abstract

*Staphylococcus aureus* is an opportunistic human pathogen that can frequently be found at various body locations, such as the upper respiratory tract, nostrils, skin, and perineum. *S. aureus* is responsible for causing a variety of conditions, which range from minor skin infections and food poisoning to life-threatening sepsis and endocarditis. Furthermore, *S. aureus* has developed resistance to numerous antimicrobial agents, which has made treatment of *S. aureus* infections difficult. In the present study, we examined lifestyle factors that could increase the likelihood of *S. aureus* carriage, the overall prevalence of *S. aureus*, as well as assessed the antibiotic resistance profiles of the *S. aureus* isolates among a population of college students. Five hundred nasal samples were collected and analyzed *via* selective growth media, coagulase and protein A testing, as well as polymerase chain reaction and DNA sequencing. One hundred four out of the 500 samples collected (21%) were identified as containing *S. aureus*. The *S. aureus* isolates were resistant to penicillin (74%), azithromycin (34%), cefoxitin (5%), ciprofloxacin (5%), tetracycline (4%), and trimethoprim (1%), but sensitive to gentamicin and rifampin. Lastly, we identified several lifestyle factors (*i.e.*, pet exposure, time spent at the university recreational facility, musical instrument usage, and tobacco usage) positively correlated with *S. aureus* nasal colonization.

## Introduction

The opportunistic pathogen, *S. aureus*, colonizes approximately 20% - 40% of the general population, while 20% - 60% are intermittent carriers (Kluytmans et al., 1997; Kuehnert et al., 2006; Chambers and DeLeo, 2009; Sakwinska et al., 2009; Warnke et al., 2014). Nasal carriage of *S. aureus* varies depending upon the population studied, method of sampling, and identification techniques utilized (Kluytmans et al., 1997). Usually*, S. aureus* results in minor skin infections but has the propensity to develop into life-threatening conditions. Studies have shown that individuals who have had recent surgery, experienced invasive medical device usage, or exhibit a weakened immune system, are much more susceptible to *S. aureus* infections (Kluytmans et al., 1997; Lowy, 1998). Hematogenous dissemination of *S. aureus* can result in endocarditis, osteomyelitis, septic arthritis, or epidural abscesses (Archer, 1998).

Previous reports have shown that asymptomatic individuals who carry *S. aureus* are at a greater risk for infection (Miles et al., 1944; Kluytmans et al., 1997; von Eiff et al., 2001) and can serve as a reservoir of transmittance via direct contact or through fomites (Lu et al., 2005; Jaradat et al., 2020). *S. aureus* is difficult to treat due to its high propensity to develop antibiotic resistance (Foster, 2017). In the decades following its discovery, methicillin resistant *S. aureus* (MRSA) (Jevons, 1961), which is resistant to β-lactam antibiotics, has been frequently implicated in healthcare-associated infections (Chambers and DeLeo, 2009; Krishnamurthy et al., 2014). However, studies have shown the emergence of MRSA transmission and infection in those without previous health-care contact, which is referred to as community-associated MRSA (CA-MRSA) (DeLeo et al., 2010; Udo, 2013). Therefore, there is a need to identify potential life-style factors that can contribute to *S. aureus* carriage, which could be used to mitigate transmission and subsequent infection.

The aim of the present study was to examine the prevalence of *S. aureus* among college-aged students and assess several life-style factors that could contribute to its nasal carriage. Among the *S. aureus* isolates we obtained, we assessed their antibiotic resistance to penicillin, azithromycin, ciprofloxacin, cefoxitin, tetracycline, trimethoprim, gentamicin, and rifampin.

## Materials and methods

### Questionnaire of potential risk factors

The participants’ risk factors were collected using a standardized paper-based questionnaire (**Supplemental Material**). The following potential risk factors for *S. aureus* colonization were assessed: public transportation usage, tobacco usage, shaving and makeup application, piercings and tattoos, number of bathroom mates, sleep, sheet changes, pet exposure, type of housing, gym usage, athletics, musical instrument usage, age, gender, race, antibiotic exposure, healthcare exposure, and previous exposure to *S. aureus.*

### Sample collection

In accordance with the University of Hartford Institutional Review Board, nasal swabs were collected from 500 undergraduate students at the University of Hartford between September 2019 and December 2019. Samples were obtained by inserting the tip of a sterile swab (BD BBL™ CultureSwab collection and transport system) along the inner edge of the participant’s nostril. The swab was then rotated while continuously circulating along the nasal vestibule for 5 sec and then this was repeated in the second nostril using the same swab (Warnke et al., 2014). The transport swab was then labeled and sealed until it was used to inoculate enrichment media.

### *Staphylococci* enrichment

Swabs were placed in 14 ml snap-cap tubes containing 3 ml of staph broth (BD Difco) and incubated at 35° C for 20 – 24 h, shaking at 225 rpm. The following day, cultures were streaked onto mannitol salt agar (MSA) plates (Oxoid) and grown overnight at 35° C. Plates that were negative for mannitol fermentation were discarded. From the plates that turned yellow, a single colony was selected and re-streaked for isolation onto a subsequent MSA plate (if multiple colony types appeared on a single plate, each was re-streaked) and incubated overnight. The following day, a single well-isolated colony was used to make a frozen stock of the bacterial strain, as described below.

### Glycerol storage

A single isolated colony was grown overnight in approximately 3 ml of trypticase soy broth (Remel) at 35° C shaking at 225 rpm. The following day, 500 μl of the culture was added to 500 μl of 50% glycerol in a 2 ml cryovial tube, vortexed for 30 sec, and stored at -80° C.

### Coagulase/protein A testing

Strains were streaked for isolation onto trypticase soy agar (TSA) plates from the frozen stocks and incubated overnight at 35° C. The Staphaurex™ Latex Agglutination Test was used according to the manufacturer’s instructions.

### Kirby Bauer disk diffusion susceptibility test

Samples were streaked for isolation onto TSA plates and incubated at 35° C overnight. The following morning, three to four bacterial colonies were suspended in sterile PBS (Gibco) in an autoclaved 10 mm x 100 mm glass tube. The suspension was then compared to a 0.5 McFarland standard. If needed, additional cells were added to achieve a similar turbidity. A sterile swab was then dipped into the suspension and used to inoculate a Mueller Hinton agar plate (Oxoid). The plates were allowed to dry for 5 to 15 min. The following antibiotic disks were used: (Oxoid Antimicrobial Susceptibility Test Disks) penicillin G (10 IU), cefoxitin (30 µg), tetracycline (30 µg), trimethoprim (5 µg), azithromycin (15 µg), ciprofloxacin (5 µg), gentamicin (10 µg), and rifampin (5 µg). Each disk was placed on the plate equidistant from each other using sterile forceps. Plates were then incubated at 35° C for 16 - 18 h (24 h for cefoxitin). Once incubation was complete, the diameter of the zones of inhibition were measured in mm. The values were compared to the Clinical and Laboratory Standards Institute supplement M100 (CLSI, 2021) chart to determine sensitivity or resistance. Zone breakpoints for determining resistance were as follows: penicillin (≤ 28 mm), trimethoprim (≤ 10 mm), tetracycline (≤ 14 mm), cefoxitin (≤ 24 mm), azithromycin (≤ 13 mm), gentamicin (≤ 12 mm), ciprofloxacin (≤ 15 mm), and rifampin (≤ 16 mm). The assay was performed in triplicate and the average zone of inhibition was used to initially screen for resistance.

### DNA isolation

Strains were streaked for isolation onto TSA plates and incubated at 35° C overnight. One-quarter loopful (10 μl Inoculating loops) of freshly streaked bacterial colonies were suspended in a 1.5 ml microcentrifuge tube containing 200 µl of 5% Chelex (BIO-RAD) in TE buffer. The tubes were vortexed so that no clumps were present and placed in a 100° C heating block for 10 min. Samples were vortexed for 30 sec, placed on ice, then subsequently centrifuged at 15,000 rcf for 10 min. 100 µl of the supernatant were transferred into a new 1.5 ml microcentrifuge tube. DNA concentrations and purity were assessed using a Nanodrop (DeNovix DS-11FX) and samples were then subsequently stored at -20° C.

### Polymerase chain reaction

Each reaction mixture contained < 250 ng of DNA, 1x GoTaq^®^polymerase (Promega), 0.4 µM of each primer in a final volume of 25 µl. The amplification conditions were as follows: (i) 5 min at 95°; (ii) 30 cycles of 30 s at 95° C, 30 s at X (**Table S1**), and 30 s for every 0.5 kb of amplicon (**Table S1**) at 72° C; (iii) 5 min at 72° C. The PCR products were separated on a 0.8% agarose (American Bioanalytical) gel in TBE to verify amplification.

### DNA sequencing

PCR amplicons were purified using the QIAquick PCR Purification Kit (QIAGEN) according to the manufacturer’s instructions. The samples were stored at - 20° C until used for sequencing. The 27F primer was used to obtain a partial sequence of the 16S rRNA gene. Sequences of at least 550 bp, which encompass the V1 – V3 regions of the gene, were compared with the NCBI database using BLASTN (Altschul et al., 1990). Sanger sequencing reactions were completed at the W. M. KECK Biotechnology Resource Laboratory at the Yale University School of Medicine, in New Haven, CT. Reactions contained 500 ng of template, 2 μl of 4 μM primer, in a total volume of 18 μl.

### Data analysis

The python scipy library was used to calculate the Fisher Exact test null-hypothesis and probability (p-value) of observing the distribution for a non-associated lifestyle factor (Virtanen et al., 2020). Here, for example, a small p-value such as *p* = 0.0035 means there is a 0.03% chance of observing the distribution at random and a 99.7% chance there is an association with the factor and *S. aureus* carriage. Since we used survey data, which possesses inherent self-reporting bias, we used a p-value cutoff of 0.15 as the other 85% of the data lacks evidence for any association. Accounting for social desirability in the survey was not possible due to validation costs related to the determination of each of the variables. Instead, we report results with the understanding that some social desirability bias is inherent in the results and that variables like “using tobacco” are under reported (Althubaiti, 2016). We also calculated and report the relative risk for the most significant associations with *S. aureus.* For additional details, see **Supplemental Materials**: Data Analysis.

## Results

### *Staphylococci* identification

To determine the number of individuals in our study who were carrying *S. aureus*, after an overnight enrichment in staph broth, nasal swab samples were streaked onto mannitol salt agar plates to screen for *S. aureus*. Of the 500 samples collected, 140 were positive for mannitol fermentation as indicated by the change in media color from red to yellow (**Table 1**). We next wanted to confirm the suspected identity of these isolates as *S. aureus* by determining the presence of coagulase and/or protein A using a latex agglutination assay. Surprisingly, only 103 of the 140 isolates were positive for coagulase and/or protein A (**Table 1**). To verify the identity of the 103 isolates as *S. aureus* and to determine the identity of the coagulase-negative staphylococci (CoNS), we PCR amplified and partially sequenced the 16S rRNA gene for each of the 140 isolates (**Table 1**). Molecular identification verified the 103 coagulase and/or protein A-positive isolates as *S. aureus* as well as identified one of the coagulase and/or protein A-negative isolates as *S. aureus* (**Table 1**). Therefore, 104 out of the 500 individuals sampled (21%) were positive for *S. aureus.* The remaining 36 isolates were identified as the following CoNS: *Staphylococcus capitis*, *Staphylococcus cohnii*, *Staphylococcus haemolyticus*, *Staphylococcus pasteuri*, *Staphylococcus saprophyticus*, *Staphylococcus warneri*, and *Staphylococcus xylosus* isolates (**Table 1**).

**Table 1:** Identification of the 140 Halotolerant – Mannitol Fermenting Isolates.

Coagulase and/or protein A	Molecular identification	Number of isolates
Positive	*S. aureus*	103
Negative	*S. aureus*	1
	*S. capitis*	1
	*S. cohnii*	3
	*S. haemolyticus*	4
	*S. pasteuri*	2
	*S. saprophyticus*	19
	*S. warneri*	5
	*S. xylosus*	2

### Antimicrobial resistance among *S. aureus* isolates

To determine the antimicrobial resistance profiles among the *S. aureus* isolates, we utilized the Kirby-Bauer disk diffusion susceptibility test to first screen for resistance to penicillin, cefoxitin, tetracycline, and trimethoprim. Cefoxitin is used to detect methicillin resistance as it is a better inducer of the methicillin resistance gene, *mecA* (CLSI, 2021). Resistance was then confirmed *via* PCR and sequencing of genes known to confer resistance to penicillin (*blaZ*), cefoxitin (*mecA*), tetracycline (*tetM*, *K*, *O*, *L*), trimethoprim (*dfrS1*), ciprofloxacin (*norA*), and azithromycin (*ermA, ermC*, *msrA*) (**Table 2**). Of the 104 *S. aureus* isolates, 74%, 5%, 4%, and 1% were resistant to penicillin, cefoxitin, tetracycline, and trimethoprim, respectively (**Table 3**). Of the five MRSA isolates, four were identified as the USA300 clone of CA-MRSA.

**Table 2:** Antimicrobial Resistance Gene(s) Detected Among the 104 *S. aureus* Isolates.

Gene(s)	Number of isolates
*blaZ*	63
*blaZ, ermA*	9
*blaZ*, *mecA*	2
*blaZ, msrA*	2
*norA*	2
*ermA*	1
*ermC*	1
*msrA*	1
*blaZ*, *mecA, msrA, norA*	1
*blaZ, ermA, norA*	1
*blaZ, ermC*	1
*blaZ*, *tetM*	1
*tetM, ermC*	1
*tetK, msrA*	1
*mecA*, *dfrS1, ermC, norA*	1
*blaZ*, *mecA*, *tetK, ermA*	1

An azithromycin resistance gene was not identified in 14 of the 35 resistant strains. *ermA*, *ermC*, and *msrA*, are the most prevalent, but several mechanisms exist (Bishr et al., 2021; Miklasińska-Majdanik, 2021).

**Table 3:** Antimicrobial Resistance Detected Among the 104 *S. aureus* Isolates.

Antimicrobial	Number of isolates resistant	Percentage
Penicillin	77	74.04
Azithromycin	35	33.65
Cefoxitin	5	4.81
Ciprofloxacin	5	4.81
Tetracycline	4	3.85
Trimethoprim	1	0.96
Gentamicin	0	0.0
Rifampicin	0	0.0

### Lifestyle factors that contribute to *S. aureus* carriage

Several lifestyle factors were examined *via* a questionnaire to determine if there was an association between an activity and *S. aureus* nasal carriage. Students with pet exposure were 38% (FET, *p* = 0.08) more likely to carry *S. aureus*. Students who attended a recreational facility more than once a week for at least 30 min were 35% (FET, *p* = 0.06) more likely to be colonized by *S. aureus*. Students who play a musical instrument were 37% (FET, *p* = 0.08) more likely to carry *S. aureus* than those who do not. Lastly, tobacco product usage was the most significant factor detected in this study, revealing that smokers were 78% (FET, *p* = 0.005) more likely to carry *S. aureus* compared to non-smokers.

## Discussion

In the current study, we evaluated the prevalence of *S. aureus* for 500 college-aged students, aged 17-25 years, that attended the University of Hartford (West Hartford, CT) in fall of 2019. Of the *S. aureus* isolates, we determined their antibiotic resistance to azithromycin, ciprofloxacin, cefoxitin, tetracycline, trimethoprim, gentamicin, and rifampin. Finally, we assessed various lifestyle factors that we believed could increase the likelihood of *S. aureus* carriage.

Recent publications report high rates of *S. aureus* nasal carriage among healthcare workers (80.3%) (Rukan et al., 2021) and individuals with atopic dermatitis (69.8%) (Blicharz et al., 2020). These populations are either at a higher risk for *S. aureus* exposure or potentially have compromised immune systems, which could more easily permit *S. aureus* carriage. Recent studies investigating the prevalence of *S. aureus* in cohorts of university students report nasal carriage rates ranging from 10% (Stancu et al., 2020) to 33% (Morita et al., 2007). Our assumption is that colonization rates among populations that have increased sensitivity to *S. aureus* colonization or are thought to be more regularly exposed to it, most likely have higher nasal carriage rates. *S. aureus* nasal carriage is often reported between 20-30% for the general population (Kavanagh et al., 2018) and our data suggests that colonization rates for University of Hartford students appear on the lower end of the spectrum (21%). We contend that the cohort we studied were less sensitive to *S. aureus* colonization because they are younger, ostensibly healthy individuals, and were generally not exposed to healthcare facilities.

Several factors, such as geographic location, age, body site sampled, identification techniques utilized, and sample size of the study, may contribute to the large variation in *S. aureus* colonization rates reported among studies. For example, mannitol salt agar was originally developed in the 1940s as a rapid tool to identify *S. aureus* in a clinical setting (Chapman, 1945). Phenotypic tests such as this are still used in developing countries where resources are limited. However, studies have revealed that some mannitol-fermenting CoNS can be misidentified as *S. aureus* when solely relying on this medium (Martinez et al., 1992; Merlino et al., 1996; Kawamura et al., 1998; Zadik et al., 2001; Ayeni and Odumosu, 2016; Ayeni et al., 2017; Thakur et al., 2017). Our work reinforces the results of those previous studies, as 36 of our 140 mannitol-positive isolates were indeed CoNS. The ramifications of relying on MSA alone are twofold: studies assessing *S. aureus* prevalence within the population could have numerous false positives and the current clinical relevance of CoNS could be undervalued due to misidentification. Consequently, certain species of CoNS are a larger health concern than previously thought (Heilmann et al., 2019). For example, *S. haemolyticus S. capitis*, and *S. saprophyticus*, have been implicated in bacteremia, neonatal sepsis, and urinary-tract infections, respectively (Becker et al., 2014; Cameron et al., 2015; Argemi et al., 2019). While our data strengthens the notion that certain members of CoNS can be misidentified as *S. aureus* in the clinical setting, CoNS are most likely more prevalent within the population than what was detected by our study. One study detected *S. haemolyticus*, *S. capitis*, *S. hominis*, *S. warneri*, and *S. lugdunensis* in 44%, 41%, 41%, 32%, and 27% of patients sampled, respectively (Kaspar et al., 2016). The higher prevalence of CoNS found in their study is most likely due to the more vigorous enrichment procedures they performed post sampling. The importance of these members of the nasal microbiota should not be overlooked due to their capacity to serve as opportunistic pathogens (Heilmann et al., 2019), reservoirs of antibiotic resistance for more pathogenic organisms (*i.e., S. aureus*) (Xu et al., 2018), or conversely, some of the nonpathogenic CoNS could benefit human health by potentially serving as antagonists for *S. aureus* colonization (Sakr et al., 2018).

Despite the re-emergence of penicillin susceptible *S. aureus*, methicillin resistant *S. aureus* (MRSA) is still a major global health concern, as approximately 53% of *S. aureus* clinical isolates are resistant to methicillin in the U.S. (Lee et al., 2018). While MRSA tends to cause serious complications such as bloodstream infections, pneumonia, or surgical site infections among those who acquire it in healthcare settings, CA-MRSA is becoming a growing concern (Hiramatsu et al., 2002; DeLeo et al., 2010). For example, CA-MRSA is predominately responsible for most skin and soft tissue infections (SSTIs) presenting in the U.S. (Moran et al., 2006; Talan et al., 2011). The CDC reports that 2% of the general population carry MRSA as part of their normal flora and it has been suggested that MRSA is replacing methicillin-susceptible *S. aureus* (MSSA) among our natural flora (Hiramatsu et al., 2002), which has potentially led to a higher prevalence of CA-MRSA infections (Udo, 2013; Lee et al., 2018). In our study, among our sampled population, 1% (5 out of 500) possessed MRSA, which comprised approximately 5% of the *S. aureus* isolates, which is consistent with previous studies that detected 0.8% - 9.2% of study participants carried MRSA (Suggs et al., 1999; Creech et al., 2005). Additionally, we were able to determine that four of the five MRSA isolates in our study were the highly virulent and transmissible CA-MRSA clone, USA300 (Pan et al., 2005). Monitoring CA-MRSA and identifying transmission factors is an important step in controlling the rapid dissemination of CA-MRSA among the population. It is believed that CA-MRSA is more easily transmitted than HA-MRSA and possesses greater pathogenic capability (Hiramatsu et al., 2002; DeLeo et al., 2010).

Previous studies have demonstrated various lifestyle activities that can increase an individual’s likelihood of carrying *S. aureus*. Our analyses revealed that pet exposure, time spent at the recreational facility, musical instrument usage, and tobacco usage, led to an increase in *S. aureus* nasal carriage. While human *S. aureus* carriage rates are between 20-30%, rates within populations of livestock can be exceedingly higher, with up to 90% of chickens, 42% of pigs, 29% of sheep and 35% of cattle being carriers (Haag et al., 2019). Rates of *S. aureus* prevalence are markedly lower in domesticated animals, with 17.5% (Chai et al., 2021) to 19.17% of cats (Bierowiec et al., 2016) and between 4.9% (Sahin-Tóth et al., 2021) and 20% (Chai et al., 2021) of dogs being carriers. Evidence supports the bidirectional transfer of *S. aureus* from humans to pets *via* licking, biting and other types of human-pet interactions (Davis et al., 2012). Our survey questions did not interrogate what type(s) of pets our participants were exposed to, but our assumption was that any increased exposure to either livestock or domestic animals would predict *S. aureus* carriage.

Previous studies have reported that both MSSA and MRSA strains have repeatedly been found on surfaces within exercise facilities with MSSA rates varying from 10% (Markley et al., 2012) to 78.3% (Bilung et al., 2018) to 90.6% (Mukherjee et al., 2016) and MRSA rates at 37.5% (Mukherjee et al., 2016). Since, recreation facilities possess numerous high-contact fomites, such as multi-user equipment, locker rooms, and water fountains, it is not surprising that *S. aureus* nasal carriage correlated with frequency of gym use as suggested by our data. It is likely that fomite transmission is the mechanism of transmission for students in our study at the on-campus recreation center or at an off-campus facility.

We also observed that musical instrument usage is correlated with higher *S. aureus* nasal carriage. Previous work has found that *S. aureus* can survive on clarinets for up to 5 days (Marshall and McBryde, 2014) and it has been suggested that bacteria remain viable on frequently touched regions of string instruments (Gambichler et al., 2004). Our survey asked participants to identify whether they made facial contact with their instrument. Our findings suggest that both facial and non-facial contact with musical instruments positively predicts *S. aureus* carriage, most likely due to some degree of fomite transmission.

Lastly, we revealed that tobacco usage was the strongest positive predictor for *S. aureus* nasal carriage in our study. Cigarette smoke has been found to alter buccal cavity microbiota by attenuating tobacco-sensitive bacterial growth and promoting the growth of tobacco-resistant bacteria (Grine et al., 2019). Specifically, tobacco tar-resistant *S. aureus* was found to be enriched in the buccal cavity of smokers and caused the increase in expression of cytokines (Fujiki et al., 2004). Other studies revealed that tobacco usage alters biofilm formation (Kumar et al., 2011) and heavily alters the composition of the oral microbiome (Pushalkar et al., 2020). Our survey asked students to identify the frequency by which they used tobacco products but did not ask if those products were single use (cigarettes/cigars) or re-usable (electronic cigarettes). Previous studies suggest that electronic cigarettes are a harbor for bacteria (Lee et al., 2019) and potentially could act as a reservoir for *S. aureus* transmission. Our rational as to why tobacco usage is such a strong predictor of *S. aureus* nasal carriage is due to two phenomena; first we entertain the possibility at least some of our tobacco using participants were using electronic cigarettes, which can act as a reservoir for *S. aureus* transmission and secondly due to tobacco’s effects on both the nascent nasal microbiota (Jaspers, 2014; Pfeiffer et al., 2021) and immune system of the user (Martin et al., 2016; Qiu et al., 2017). We contend that these two factors might work in a synergistic manner that leads to the disruption of the nascent nasal microbiota. This loss of competitive exclusion then allows for opportunistic pathogens, such as *S. aureus*, to colonize and/or increase in relative abundance. For example, previous studies have suggested that other members of the nasal microbiota may outcompete or inhibit the growth of *S. aureus* (Sakr et al., 2018).

Our data suggest that pet exposure, time spent at a recreational facility, musical instrument usage, and tobacco usage, can lead to a heightened likelihood of carrying *S. aureus*. Of these, pets and high-contact fomites found at the recreational facility may directly act as a bi-directional reservoir that allows for the transmission of *S. aureus* between individuals. To a lesser extent, instruments, and tobacco (*i.e.*, cigarettes and electronic cigarettes), could also serve as a bi-directional reservoir if these fomites are shared with others. It has been reported that up to 60% of individuals are transiently colonized by *S. aureus* (Kluytmans et al., 1997; Fournier and Philpott, 2010). It is plausible that the latter mentioned fomites serve as a reservoir that facilitates one’s own re-acquisition of *S. aureus.*

*S. aureus* is a continued global health concern that not only impacts those in healthcare facilities but also individuals within the community due to its multitude of virulence factors and propensity to acquire antibiotic resistance. Continued investigation into potential transmission mechanisms of *S. aureus* could help mitigate the spread of *S. aureus*, while ongoing surveillance of the ever-changing antimicrobial resistance landscape of *S*. *aureus* will be essential to guide empiric antimicrobial therapy. However, the key to controlling *S. aureus* colonization and subsequent pathogenesis may be found in factors that shape the nasal microbiota and subsequently antagonize or potentially leave the door open for *S. aureus* colonization.

## Data availability statement

The sequencing data is available via GenBank Accession Numbers OQ626021 – OQ626160. The datasets presented in this study can be found in the article/**Supplementary Material**.

## Ethics statement

This study was approved by the Institutional Review Board (IRB) of the University of Hartford. The patients/participants provided their written informed consent to participate in this study.

## Author contributions

SC, JG, KM, MA, DT, YA-A, AP, TB, AS contributed to the conception and design of the study. SC, JG, KM, MA, DT, YA-A, AP, AS, OR performed experiments and collected data. TB performed the statistical analysis. SC, JG, AB, AS, TB, AS wrote sections of the manuscript. All authors contributed to the article and approved the submitted version.
