# Supplementary material for: Prevalence and antibiotic resistance of Staphylococcus aureus associated with a college-aged cohort: life-style factors that contribute to nasal carriage
Source: Front Cell Infect Microbiol. 2023 Jun 27;13:1195758. doi: 10.3389/fcimb.2023.1195758 (PMC10333693; doi:10.3389/fcimb.2023.1195758)
Supplement: Supplementary file 1 [file DataSheet_1.docx]

**Supplemental Data Analysis**

Data analysis was conducted by integrating survey questions with the biological tests (Materials and Methods). The integrated table (**Supplemental File 1:** S.aureus-Data Set 10.4.21.xlsx) was converted to tab-separated file format (**Supplemental File 2:** staph_project_v1.4.tsv). Every data column had valid numerical results based on the survey design and biological testing procedures. To automate the data processing and significance tests, a python3 script was written and provided for validation (**Supplemental File 3:** staph_analysis.py). First the completed data table is opened (**Supplemental File 2**) and then data transformations of the life-style (independent) variables are calculated as follows:

The **Housing** variable was transformed to **YES** if either the **Dorm** or **On-campus** option was selected and **NO** otherwise.

The variable **Gym_Time** was transformed to **YES** when students selected any survey option other than 0-1 times a week of 30 minutes of exercise, and **NO** otherwise.

The variable **Sheet_Change** we transformed the total semester count to be **YES** when the student reported as changing any more than 2 times in the 14-week semester and **NO** otherwise.

The variable **Weekday_Sleep** we considered more than 7 hours a **YES** and **NO** otherwise along with the variable **Weekend_Sleep_Offset** of more than 1 to be a **YES** and **NO** otherwise. This followed from common sleep studies that provide evidence of 8 or more hours of sleep as defining a well-rested subject (Watson et al. 2015).

*S. aureus* was then treated as a dependent variable and the life-style factors (which are the independent variables) were then tested for significance using Fisher’s Exact test to find the probability of observing a more extreme distribution. Our tests all followed the table form:

|  | **Variable=YES** | **Variable=NO** | *Row Total* |
| --- | --- | --- | --- |
| ***S.aureus*^+^** | **a** | **b** | **a+b** |
| ***S.aureus*^-^** | **c** | **d** | **c+d** |
| *Column Total* | **a+c** | **b+d** | **a+b+c+d=n** |

The Fisher Exact Test p-value for the test is calculated as:

**(a+b)!(c+d)!(a+c)!(b+d)!/(a!b!c!d!(a!+b!+c!+d!)!)**

Using a cutoff of 0.15 we observed four significant variables: **Tobacco_Usage**=0.005, **Musical_Instrument_Usage**=0.08, **Gym_Time**=0.06, **Pet_Exposure**=0.08.

| **Tobacco_Usage** | **YES** | **NO** | *Row Total* |
| --- | --- | --- | --- |
| ***S. aureus***(+) | **27** | **76** | **103** |
| ***S. aureus***(-) | **58** | **339** | **397** |
| *Column Total* | **85** | **415** | **500** |

| **Musical_Instrument** | **YES** | **NO** | *Row Total* |
| --- | --- | --- | --- |
| ***S. aureus***(+) | **28** | **75** | **103** |
| ***S. aureus***(-) | **80** | **317** | **397** |
| *Column Total* | **108** | **392** | **500** |

| **Gym_Time** | **YES** | **NO** | *Row Total* |
| --- | --- | --- | --- |
| ***S. aureus***(+) | **73** | **30** | **103** |
| ***S. aureus***(-) | **246** | **151** | **397** |
| *Column Total* | **319** | **181** | **500** |

| **Pet_Exposure** | **YES** | **NO** | *Row Total* |
| --- | --- | --- | --- |
| ***S. aureus***(+) | **78** | **25** | **103** |
| ***S. aureus***(-) | **270** | **127** | **397** |
| *Column Total* | **348** | **152** | **500** |

**Calculation of relative risk**

[www.cdc.gov/csels/dsepd/ss1978/lesson3/section5.html](http://www.cdc.gov/csels/dsepd/ss1978/lesson3/section5.html)

**Risk of association due to tobacco**

Risk of SA+ among those using tobacco is 32% (27/85)

Risk of SA+ among those not using tobacco is 18% (76/415)

The relative risk for SA+ given tobacco usage is 32% / 18% = **1.78**

**Risk of association due to musical instrument usage**

Risk of SA+ among those using an instrument is 26% (28/108)

Risk of SA+ among those not using an instrument is 19% (75/392)

The relative risk for SA+ given instrument usage is 26% / 19% = **1.37**

**Risk of association due to gym usage**

Risk of SA+ among those using a recreational facility is 23% (73/319)

Risk of SA+ among those not using a recreational facility is 17% (30/181)

The relative risk for SA+ given recreational facility usage is 23% / 17% = **1.35**

**Risk of association due to pet exposure**

Risk of SA+ among those exposed to pets is 22% (78/348)

Risk of SA+ among those not exposed to pets is 16% (25/152)

The relative risk for SA+ given pet exposure is then 22% / 16% = **1.38**

Watson N.F., Badr M.S., Belenky G., Bliwise D.L., Buxton O.M., Buysse D., Dinges D.F., Gangwisch J., Grandner M.A., Kushida C., Malhotra R.K., Martin J.L., Patel S.R., Quan S.F., Tasali E. Recommended Amount of Sleep for a Healthy Adult: A Joint Consensus Statement of the American Academy of Sleep Medicine and Sleep Research Society. Sleep. 2015 Jun 1;38(6):843-4. doi:10.5665/sleep.4716. PMID: 26039963; PMCID:PMC4434546.
