# Supplementary material for: Prevalence and antibiotic resistance of Staphylococcus aureus associated with a college-aged cohort: life-style factors that contribute to nasal carriage
Source: Front Cell Infect Microbiol. 2023 Jun 27;13:1195758. doi: 10.3389/fcimb.2023.1195758 (PMC10333693; doi:10.3389/fcimb.2023.1195758)
Supplement: Supplementary file 3 [file DataSheet_3.pdf]

Supplemental File 2

Sample_Number	Race	College	Sports_and_Equipment	Staph_Exposure	Musical_Instrument_Usage	Housing	Gym_Time	Sheet_Change	Gender	Tobacco_Usage	Weekday_Sleep	Weekend_Sleep_Offset	Healthcare_Exposure	Antibiotics_Exposure	Bathroom_Mates	Piercings_and_Tats	Shave_or_Makeup	Age	Public_Transport_Usage	Pet_Exposure
1	White	ENHS	Equipment	NO	NO	On-Campus	195	4	F	0	5.5	3	0	3	3.5	1.5	1.5	20	0	5
2	White	ENHS	Equipment	Lived	Face	Dorm	195	3	F	0	6.5	4	0	0	7.5	0	7.5	18	0	25
3	White	BARNEY	No-equipment	NO	NO	On-Campus	225	3	M	0	7.5	2	30	1	0	0	1.5	22	0	0
4	Asian	ENHS	Equipment	NO	Non-Face	Dorm	225	3	F	0	7.5	4	3	1	3.5	1.5	5.5	20	1.5	5
5	White	ENHS	Equipment	NO	NO	On-Campus	225	4	F	0	5.5	2	10	4	3.5	7.5	1.5	19	0	25
6	White	ENHS	Equipment	NO	NO	Dorm	225	3	F	0	7.5	0	0	0	3.5	1.5	1.5	20	0	0
7	Black	A&S	Shared	NO	NO	Dorm	195	4	M	0	6.5	2	3	0	5.5	0	1.5	19	0	5
8	White	ENHS	Non-Contact	NO	NO	Dorm	75	4	F	0	8.5	2	3	1	7.5	0	0	18	0	15
9	White	A&S	Equipment	NO	NO	Dorm	195	3	F	0	5.5	0	3	2	7.5	1.5	0	19	0	5
10	White	BARNEY	Shared	NO	NO	Dorm	195	3	F	4	5.5	3	3	1	7.5	0	1.5	18	0	5
11	White	BARNEY	Shared	NO	NO	Dorm	195	3	F	0	5.5	2	0	0	3.5	0	0	18	0	0
12	White	ENHS	NO	NO	NO	Dorm	225	3	F	0	6.5	0	0	1	5.5	0	1.5	18	0	15
13	White	ENHS	No-equipment	NO	NO	On-Campus	195	2	M	4	7.5	3	3	4	5.5	0	1.5	20	0	5
14	White	A&S	Equipment	NO	Face	On-Campus	135	4	F	0	7.5	3	3	4	3.5	0	1.5	19	0	15
15	White	ENHS	Equipment	NO	NO	On-Campus	135	4	F	0	6.5	1	3	2	1.5	0	1.5	20	0	15
16	White	ENHS	Shared	NO	NO	Off-Campus	75	4	F	0	7.5	0	30	2	1.5	0	5.5	22	0	30
17	White	BARNEY	Equipment	NO	NO	On-Campus	195	2	M	0	6.5	1	3	0	1.5	0	1.5	21	0	5
18	White	HARTT/HILLYER	No-equipment	NO	NO	Dorm	195	4	M	10	6.5	3	3	1	1.5	1.5	0	19	0	5
19	White	CETA	Equipment	NO	NO	Dorm	195	1	M	1	6.5	0	0	1	5.5	0	1.5	18	0	0
20	Other	CETA	Equipment	NO	NO	Dorm	225	4	M	0	7.5	2	0	2	5.5	0	1.5	18	0	15
21	Black	BARNEY	Shared	NO	NO	Dorm	135	3	M	1	6.5	0	3	2	5.5	1.5	3.5	18	0	5
22	Black	HARTT/HILLYER	Equipment	NO	NO	Dorm	225	1	M	10	6.5	3	3	4	7.5	0	0	18	5.5	0
23	White	ENHS	Non-Contact	Had-Lived	NO	Dorm	225	4	F	0	6.5	3	3	2	1.5	0	3.5	21	0	15
24	Black	A&S	Non-Contact	NO	NO	Dorm	225	4	F	0	5.5	3	3	1	3.5	1.5	1.5	19	5.5	5
25	White	A&S	Non-Contact	NO	NO	On-Campus	225	4	F	0	6.5	1	3	2	3.5	1.5	1.5	20	1.5	15
26	White	ENHS	Equipment	NO	NO	Dorm	195	3	M	0	6.5	0	3	1	5.5	0	1.5	18	0	5
27	White	BARNEY	Equipment	NO	NO	Dorm	195	2	M	4	7.5	2	3	1	7.5	0	1.5	18	0	0
28	Black	ENHS	Non-Contact	NO	NO	On-Campus	195	4	M	0	5.5	4	3	1	3.5	0	1.5	20	0	5
29	White	CETA	Shared	NO	Non-Face	Dorm	135	3	M	0	6.5	2	0	0	5.5	0	1.5	18	0	5
30	Black	CETA	Non-Contact	NO	NO	Dorm	75	4	M	0	5.5	1	10	1	7.5	0	0	18	3.5	0
31	White	BARNEY	NO	NO	NO	Dorm	135	3	M	0	6.5	2	30	2	7.5	0	3.5	20	1.5	0
32	Latino	BARNEY	Shared	NO	Face	Dorm	195	4	M	0.5	4.5	0	10	1	3.5	0	0	18	0	30
33	White	BARNEY	Shared	NO	NO	On-Campus	195	4	M	0	7.5	3	0	0	3.5	4	1.5	20	0	25
34	White	BARNEY	NO	NO	NO	On-Campus	15	1	M	0	6.5	3	3	0	3.5	0	1.5	20	0	5
35	White	ENHS	NO	NO	NO	Off-Campus	75	4	M	0	4.5	1	30	0	3.5	0	1.5	22	0	30
36	White	A&S	Non-Contact	NO	NO	On-Campus	135	4	F	0	4.5	2	3	3	3.5	0	1.5	21	0	5
37	White	A&S	NO	NO	Non-Face	On-Campus	135	4	M	0	5.5	3	3	0	3.5	0	3.5	20	0	5
38	White	A&S	Non-Contact	NO	NO	On-Campus	225	4	F	0	6.5	3	3	2	1.5	1.5	3.5	20	0	5
39	White	A&S	NO	NO	NO	House	15	4	M	0	7.5	0	0	0	3.5	0	1.5	22	0	5
40	White	BARNEY	NO	NO	NO	House	75	4	M	10	7.5	3	0	1	3.5	1.5	1.5	20	0	5
41	White	BARNEY	NO	NO	NO	On-Campus	75	4	M	10	6.5	2	0	3	1.5	0	1.5	20	0	5
42	White	A&S	NO	NO	NO	Off-Campus	75	4	M	10	5.5	2	3	0	3.5	0	1.5	22	0	5
43	Black	A&S	NO	Had	NO	On-Campus	15	4	F	0	6.5	0	0	3	3.5	0	1.5	20	0	0
44	Black	A&S	NO	NO	NO	Dorm	135	2	M	0	7.5	4	3	0	3.5	0	0	20	3.5	0
45	White	ENHS	Equipment	NO	NO	On-Campus	225	4	F	0	8.5	0	0	0	3.5	1.5	1.5	21	0	0
46	White	CETA	Equipment	NO	NO	On-Campus	225	4	F	0	8.5	0	3	2	3.5	0	1.5	22	0	0
47	White	ENHS	NO	Lived	NO	On-Campus	135	3	F	0	7.5	0	3	2	1.5	0	1.5	20	0	5
48	Other	ENHS	NO	NO	Face	On-Campus	135	4	F	0	5.5	3	3	1	3.5	0	3.5	19	0	5
49	White	ENHS	Non-Contact	NO	NO	Dorm	195	2	M	10	6.5	2	3	1	7.5	0	1.5	20	0	0
50	White	ENHS	Non-Contact	NO	NO	On-Campus	135	4	F	0	5.5	2	10	4	3.5	0	3.5	21	0	5
51	White	CETA	NO	NO	NO	On-Campus	135	3	M	0	6.5	0	10	1	3.5	1.5	3.5	19	0	15
52	White	ENHS	NO	NO	NO	On-Campus	15	4	F	4	7.5	0	3	4	3.5	1.5	3.5	19	0	5
53	Latino	BARNEY	Non-Contact	NO	NO	On-Campus	225	3	F	0	7.5	3	0	1	5.5	4	1.5	19	0	5
54	Black	BARNEY	Equipment	NO	NO	Dorm	225	4	F	0	7.5	1	3	1	7.5	0	3.5	18	0	0
55	White	BARNEY	Equipment	NO	NO	Dorm	225	4	F	0	7.5	1	3	1	7.5	0	5.5	19	0	5
56	White	ENHS	NO	NO	NO	On-Campus	15	4	F	0	5.5	1	3	2	3.5	0	0	19	0	5
57	White	CETA	NO	NO	Face	On-Campus	15	2	F	0	5.5	2	0	1	3.5	1.5	1.5	19	0	5
58	White	A&S	NO	NO	NO	Dorm	75	4	F	0	7.5	1	10	3	7.5	0	1.5	19	0	0
59	White	BARNEY	No-equipment	NO	Non-Face	On-Campus	75	3	M	10	7.5	2	3	2	5.5	0	1.5	20	0	25
60	White	BARNEY	Non-Contact	NO	NO	On-Campus	75	2	M	4	7.5	2	10	2	5.5	0	3.5	20	0	25
61	Asian	BARNEY	Non-Contact	NO	NO	On-Campus	75	4	F	4	6.5	1	3	2	3.5	4	3.5	19	3.5	0
62	White	BARNEY	NO	NO	NO	On-Campus	15	4	F	0	6.5	1	0	0	3.5	1.5	7.5	19	0	15
63	White	BARNEY	No-equipment	NO	NO	On-Campus	15	3	M	0	6.5	1	3	4	5.5	0	3.5	19	0	5
64	Asian	A&S	NO	NO	Non-Face	On-Campus	15	1	F	0	5.5	2	3	0	1.5	0	1.5	19	1.5	0
65	White	CETA	NO	NO	NO	On-Campus	195	4	M	0	7.5	3	3	0	1.5	0	3.5	21	0	5
66	White	A&S	NO	NO	NO	House	75	2	M	0	7.5	3	10	3	3.5	0	1.5	19	0	30
67	White	CETA	No-equipment	NO	NO	On-Campus	195	2	F	0	7.5	1	3	0	3.5	0	3.5	19	0	5
68	White	BARNEY	No-equipment	NO	NO	Dorm	225	4	F	0	8.5	2	0	0	1.5	0	5.5	20	0	0
69	Latino	A&S	NO	NO	NO	House	75	1	M	4	4.5	1	3	2	1.5	0	0	19	0	15
70	Other	BARNEY	NO	NO	NO	On-Campus	15	4	F	1	4.5	2	0	3	3.5	0	3.5	19	1.5	0
71	Asian	ENHS	NO	NO	Non-Face	On-Campus	15	3	F	0	5.5	2	0	0	3.5	0	1.5	20	0	5
72	Latino	A&S	NO	NO	NO	On-Campus	15	4	F	0	5.5	0	0	1	3.5	4	5.5	20	0	0
73	Black	BARNEY	NO	NO	NO	On-Campus	15	4	F	0	4.5	0	0	0	3.5	0	0	19	0	0
74	Black	A&S	NO	NO	NO	On-Campus	15	3	F	0	4.5	0	3	4	3.5	1.5	3.5	19	0	0
75	Black	BARNEY	NO	NO	NO	Dorm	15	4	F	0	8.5	0	3	0	3.5	0	0	19	0	0
76	Latino	BARNEY	NO	NO	NO	Dorm	15	4	M	0	7.5	2	0	0	7.5	0	1.5	20	0	25
77	White	ENHS	No-equipment	NO	NO	On-Campus	75	3	F	0	6.5	3	10	0	3.5	1.5	1.5	21	0	0
78	Latino	CETA	NO	NO	Non-Face	On-Campus	15	4	M	0	6.5	0	3	1	3.5	0	1.5	19	0	5
79	White	HARTT/HILLYER	NO	NO	Non-Face	On-Campus	15	2	F	0	5.5	1	0	1	3.5	0	1.5	20	0	5
80	White	A&S	NO	Had	NO	Off-Campus	195	3	M	0	7.5	0	0	0	1.5	0	3.5	22	0	30
81	White	A&S	NO	NO	NO	On-Campus	15	4	F	10	6.5	2	3	4	1.5	1.5	1.5	19	0	0
82	Asian	BARNEY	Shared	NO	NO	House	135	1	M	0	5.5	0	0	0	3.5	0	5.5	19	0	0
83	Latino	CETA	No-equipment	NO	Face	Dorm	135	2	M	0	4.5	1	0	0	1.5	0	1.5	19	0	0
84	Black	A&S	No-equipment	NO	NO	Dorm	15	4	O	10	5.5	0	3	2	5.5	1.5	0	21	0	15
85	White	CETA	NO	NO	NO	Off-Campus	15	4	M	0	6.5	1	3	1	0	0	3.5	21	0	30
86	White	CETA	Equipment	NO	NO	House	75	4	F	4	6.5	2	3	2	3.5	1.5	3.5	19	0	30
87	White	CETA	NO	NO	Non-Face	On-Campus	195	2	M	1	6.5	3	0	1	3.5	0	1.5	19	0	0
88	Black	A&S	NO	NO	NO	Off-Campus	75	2	M	0	5.5	0	3	0	3.5	0	0	18	7.5	0
89	Black	HARTT/HILLYER	NO	NO	Face	Dorm	15	4	F	0	5.5	2	3	0	1.5	1.5	1.5	18	0	5
90	Asian	ENHS	NO	NO	Non-Face	House	135	3	F	4	7.5	4	0	2	1.5	0	3.5	21	0	30
91	White	A&S	Shared	NO	NO	Dorm	195	3	F	0	7.5	2	0	1	5.5	0	1.5	18	0	5
92	Black	A&S	Equipment	NO	NO	On-Campus	135	4	O	0	5.5	0	3	0	3.5	1.5	1.5	20	0	25
93	White	A&S	Non-Contact	NO	Non-Face	Dorm	75	3	F	0	6.5	0	0	2	7.5	0	3.5	19	0	5
94	White	CETA	Equipment	NO	NO	On-Campus	15	4	M	0	7.5	1	3	0	3.5	0	0	19	0	5
95	White	CETA	Non-Contact	Had-Lived	NO	On-Campus	15	3	M	0	5.5	2	10	4	3.5	0	0	19	0	5
96	Other	A&S	Non-Contact	NO	NO	House	15	3	F	0	4.5	0	3	1	1.5	0	7.5	20	0	0
97	White	CETA	NO	NO	NO	House	15	2	M	10	7.5	1	3	0	0	0	1.5	19	0	30
98	White	A&S	NO	NO	NO	On-Campus	15	4	F	0	5.5	3	3	0	3.5	1.5	1.5	20	1.5	15
99	White	A&S	Non-Contact	NO	NO	Dorm	75	3	F	0	5.5	2	0	2	3.5	0	5.5	18	1.5	5
100	Latino	A&S	NO	NO	NO	On-Campus	15	4	F	0	4.5	0	0	0	3.5	1.5	0	21	0	30
101	Asian	ENHS	NO	NO	NO	On-Campus	15	4	F	4	4.5	0	3	1	1.5	0	3.5	19	0	5
102	White	CETA	NO	NO	Face	On-Campus	75	4	M	0	8.5	1	3	1	3.5	0	1.5	21	0	0
103	White	CETA	Non-Contact	NO	Non-Face	House	75	3	M	10	7.5	1	0	0	1.5	1.5	1.5	21	0	30
104	Latino	CETA	NO	NO	NO	Dorm	15	1	M	0	6.5	0	3	2	1.5	0	1.5	21	0	0
105	Black	CETA	NO	NO	NO	Dorm	15	4	F	0	5.5	2	0	0	7.5	0	0	20	1.5	0
106	Asian	CETA	NO	NO	NO	On-Campus	135	4	F	0	6.5	3	3	2	3.5	0	1.5	20	1.5	5
107	Other	ENHS	No-equipment	NO	NO	Dorm	75	2	M	4	7.5	2	0	2	7.5	0	0	18	1.5	5
108	Black	ENHS	NO	NO	NO	Dorm	75	3	F	0	5.5	2	3	2	7.5	1.5	3.5	19	1.5	5
109	White	CETA	NO	NO	NO	House	75	4	M	0	8.5	1	0	0	0	0	3.5	22	0	25
110	White	CETA	NO	Lived	NO	On-Campus	135	4	M	10	5.5	1	3	0	1.5	4	3.5	20	0	5
111	White	CETA	NO	NO	NO	Off-Campus	195	2	F	0	5.5	2	3	0	1.5	1.5	3.5	22	0	5
112	White	HARTT/HILLYER	NO	NO	Non-Face	House	135	4	M	0	6.5	0	3	1	3.5	0	1.5	20	0	5
113	White	A&S	NO	NO	NO	On-Campus	15	1	F	0	7.5	2	3	0	1.5	1.5	1.5	19	0	5
114	Latino	A&S	Equipment	NO	NO	Dorm	195	3	F	0	6.5	2	3	1	5.5	0	3.5	18	0	0
115	White	A&S	NO	NO	NO	On-Campus	15	4	M	4	5.5	3	3	4	3.5	1.5	1.5	20	0	0
116	Black	ENHS	No-equipment	Lived	NO	Dorm	135	4	M	0	4.5	0	3	0	7.5	0	1.5	22	0	0
117	Latino	CETA	NO	NO	NO	On-Campus	15	4	F	1	5.5	0	3	3	3.5	7.5	1.5	21	1.5	30
118	White	ENHS	Non-Contact	NO	NO	Off-Campus	135	4	F	0	7.5	2	3	0	1.5	1.5	1.5	20	0	25
119	Asian	CETA	Non-Contact	NO	Non-Face	House	135	4	M	0	4.5	4	0	0	3.5	0	0	22	0	0
120	Black	CETA	Non-Contact	NO	Face	Dorm	135	3	M	0	5.5	0	3	2	7.5	0	1.5	18	0	5
121	White	ENHS	NO	NO	NO	Dorm	195	4	F	10	8.5	4	3	2	5.5	0	1.5	19	0	0
122	White	ENHS	NO	NO	NO	On-Campus	75	1	F	1	6.5	3	30	2	5.5	0	7.5	20	0	15
123	White	ENHS	No-equipment	NO	NO	House	195	4	M	0	6.5	2	3	0	1.5	0	0	22	0	30
124	Black	ENHS	NO	NO	NO	House	135	4	M	0	6.5	2	30	0	1.5	0	1.5	22	0	0
125	White	ENHS	NO	NO	NO	Off-Campus	135	3	F	0	7.5	2	0	1	3.5	0	5.5	21	0	5
126	Black	CETA	NO	NO	NO	House	75	2	M	0	6.5	1	3	0	1.5	0	0	18	0	0

127	White	A&S	NO	NO	Non-Face	Dorm	15	3	M	0	6.5	2	3	2	1.5	4	1.5	18	0	0
128	White	A&S	NO	NO	NO	House	195	4	O	0	7.5	0	0	0	5.5	1.5	1.5	19	0	30
129	White	A&S	NO	NO	Non-Face	Dorm	15	4	F	0	6.5	4	3	1	1.5	4	3.5	19	0	15
130	White	BARNEY	Non-Contact	NO	NO	Dorm	135	4	M	0	7.5	2	3	1	7.5	0	1.5	18	0	25
131	White	ENHS	NO	NO	NO	On-Campus	15	2	F	0	5.5	2	3	1	1.5	0	3.5	20	0	5
132	Black	BARNEY	NO	NO	NO	Dorm	15	3	M	0	6.5	0	3	2	3.5	0	0	18	0	0
133	Other	CETA	No-equipment	NO	NO	Dorm	75	2	M	0	5.5	3	3	2	5.5	0	1.5	19	1.5	5
134	White	ENHS	NO	NO	NO	On-Campus	75	4	M	4	7.5	2	0	1	3.5	0	1.5	19	0	5
135	Asian	CETA	NO	NO	NO	On-Campus	15	4	M	4	7.5	2	3	1	3.5	0	1.5	21	0	5
136	White	CETA	No-equipment	NO	NO	On-Campus	135	1	M	10	6.5	3	3	2	3.5	0	1.5	20	0	5
137	Black	A&S	Non-Contact	NO	NO	On-Campus	75	2	M	0	4.5	1	0	0	3.5	1.5	3.5	21	1.5	0
138	White	CETA	NO	NO	Non-Face	On-Campus	15	4	M	0	5.5	2	10	3	3.5	0	3.5	22	0	25
139	Black	A&S	Shared	NO	NO	Dorm	15	3	M	0	5.5	2	0	0	7.5	0	1.5	18	1.5	0
140	White	ENHS	NO	NO	NO	On-Campus	195	4	F	0	4.5	0	3	2	3.5	7.5	1.5	20	0	0
141	Asian	ENHS	NO	NO	NO	Dorm	75	4	F	0	5.5	2	30	1	1.5	0	3.5	21	0	15
142	Asian	ENHS	NO	NO	NO	Dorm	15	2	F	0	4.5	2	30	2	7.5	1.5	7.5	20	0	5
143	Latino	A&S	Non-Contact	NO	NO	Dorm	15	4	M	0	6.5	4	3	2	7.5	0	0	18	3.5	30
144	White	A&S	NO	NO	NO	On-Campus	135	4	M	0	6.5	0	3	0	3.5	0	1.5	21	0	25
145	White	A&S	No-equipment	Had-Lived	NO	On-Campus	75	4	F	0	5.5	1	30	2	3.5	0	3.5	20	0	5
146	Latino	A&S	NO	NO	NO	House	15	4	F	0	8.5	0	10	4	3.5	1.5	0	18	3.5	30
147	Black	CETA	No-equipment	NO	NO	House	75	4	M	0	6.5	2	3	0	1.5	0	0	18	0	0
148	White	CETA	NO	NO	NO	Dorm	75	2	M	10	4.5	2	0	2	7.5	0	1.5	18	0	5
149	White	A&S	Equipment	NO	NO	Dorm	225	4	F	0	4.5	4	10	1	3.5	0	7.5	20	0	0
150	Latino	A&S	NO	NO	Non-Face	Off-Campus	15	4	M	0	6.5	1	3	0	0	0	0	21	0	30
151	Asian	BARNEY	No-equipment	NO	NO	House	135	4	M	1	6.5	2	3	2	1.5	0	0	19	0	0
152	White	ENHS	NO	NO	NO	House	15	4	F	0	6.5	4	30	0	1.5	0	7.5	22	0	30
153	Latino	A&S	NO	NO	NO	House	135	3	F	0	5.5	2	0	1	3.5	4	5.5	19	0	5
154	Asian	ENHS	Non-Contact	NO	NO	Off-Campus	195	1	M	0	5.5	4	3	0	1.5	0	1.5	22	0	0
155	Black	CETA	NO	NO	NO	House	75	2	M	0	6.5	3	0	1	5.5	0	1.5	22	0	0
156	White	HARTT/HILLYER	NO	NO	Non-Face	Dorm	75	4	M	0	6.5	1	3	0	7.5	0	1.5	18	0	0
157	Asian	A&S	NO	NO	NO	House	15	3	F	0	7.5	1	3	1	0	1.5	3.5	20	0	0
158	Asian	BARNEY	NO	NO	NO	House	15	2	F	0	5.5	2	3	0	1.5	1.5	1.5	20	0	5
159	Other	A&S	Non-Contact	Lived	NO	House	15	3	M	10	7.5	0	0	0	5.5	0	0	20	3.5	0
160	Black	CETA	NO	NO	NO	House	15	4	F	0	5.5	0	3	0	3.5	0	1.5	20	3.5	0
161	White	A&S	NO	NO	NO	House	75	4	M	0	4.5	2	10	3	3.5	0	1.5	22	3.5	30
162	White	A&S	Non-Contact	NO	NO	Off-Campus	135	4	M	0	6.5	2	0	1	3.5	0	1.5	22	0	5
163	Latino	CETA	NO	NO	NO	House	15	0	F	0	6.5	1	0	0	0	0	1.5	18	0	5
164	White	ENHS	NO	NO	NO	Dorm	75	4	F	0.5	7.5	4	30	4	5.5	1.5	0	19	0	30
165	White	ENHS	NO	NO	NO	House	75	4	M	4	5.5	2	10	1	1.5	0	1.5	22	0	30
166	White	ENHS	NO	NO	NO	On-Campus	75	3	F	0	6.5	0	30	1	1.5	0	3.5	19	0	15
167	White	ENHS	Equipment	NO	Non-Face	Off-Campus	75	4	M	0	5.5	2	3	0	0	0	1.5	22	0	5
168	White	ENHS	NO	NO	NO	House	135	2	F	0	5.5	1	10	1	1.5	0	3.5	19	0	30
169	Black	CETA	Equipment	NO	Non-Face	Dorm	15	4	F	4	4.5	3	3	1	3.5	0	5.5	19	0	0
170	Latino	ENHS	Equipment	NO	NO	House	75	1	M	0	4.5	3	30	1	3.5	1.5	1.5	22	0	30
171	White	ENHS	NO	NO	NO	On-Campus	75	4	F	0	5.5	0	23	0	5.5	0	0	18	0	30
172	White	ENHS	No-equipment	NO	Non-Face	On-Campus	135	4	M	0	7.5	0	10	1	3.5	0	0	20	0	0
173	White	ENHS	Non-Contact	NO	NO	On-Campus	135	4	M	0	7.5	1	0	0	3.5	0	1.5	19	0	5
174	White	ENHS	NO	NO	NO	Dorm	75	3	F	0	6.5	3	3	2	7.5	0	3.5	18	0	30
175	Other	CETA	NO	NO	NO	House	15	4	F	0	5.5	0	3	1	3.5	0	0	20	0	30
176	White	ENHS	Non-Contact	NO	NO	House	75	4	F	0	6.5	1	3	0	3.5	0	0	20	0	30
177	Other	A&S	NO	NO	NO	House	15	4	F	0	7.5	0	0	0	5.5	1.5	0	22	0	30
178	Latino	ENHS	NO	NO	NO	Dorm	75	3	M	4	6.5	1	3	0	7.5	1.5	1.5	20	0	15
179	White	ENHS	NO	NO	NO	Off-Campus	15	4	F	0	4.5	2	3	0	1.5	7.5	1.5	22	0	15
180	Black	ENHS	Non-Contact	NO	NO	Dorm	75	4	F	0	6.5	4	3	0	5.5	1.5	1.5	20	0	0
181	Latino	ENHS	NO	NO	Face	Dorm	15	4	F	0	6.5	4	3	2	5.5	0	7.5	19	0	5
182	Black	A&S	NO	NO	NO	On-Campus	15	3	F	0	6.5	3	3	3	3.5	0	0	20	0	0
183	White	ENHS	NO	NO	NO	On-Campus	75	3	F	0	6.5	2	0	0	3.5	0	5.5	19	0	5
184	White	A&S	NO	NO	NO	House	135	3	F	0	6.5	2	10	1	1.5	0	3.5	22	0	25
185	Asian	ENHS	NO	NO	NO	Off-Campus	15	3	M	0	5.5	3	3	0	3.5	0	0	22	0	0
186	White	ENHS	Non-Contact	NO	NO	On-Campus	75	4	F	0	7.5	2	30	1	5.5	0	1.5	19	0	25
187	Other	ENHS	NO	Lived	NO	House	15	2	F	0	6.5	3	3	2	5.5	1.5	1.5	19	0	5
188	White	ENHS	NO	NO	NO	On-Campus	75	2	F	0	7.5	4	10	1	3.5	10	5.5	20	0	0
189	Black	A&S	NO	NO	Non-Face	House	15	4	M	0	6.5	0	30	0	1.5	0	0	22	0	0
190	White	ENHS	NO	NO	NO	House	15	1	M	0	5.5	3	3	1	5.5	0	0	22	0	5
191	White	CETA	NO	NO	NO	On-Campus	135	4	M	0	6.5	4	3	0	3.5	0	7.5	20	0	0
192	White	CETA	NO	NO	NO	Dorm	15	4	M	0	6.5	3	23	2	5.5	0	3.5	20	0	5
193	White	ENHS	NO	NO	Non-Face	House	15	2	M	0	7.5	4	3	0	5.5	0	1.5	19	0	30
194	White	ENHS	NO	NO	NO	House	15	1	M	4	6.5	3	30	0	0	0	1.5	20	0	30
195	White	ENHS	NO	NO	NO	House	15	2	F	0	7.5	4	30	0	3.5	0	5.5	20	0	30
196	Black	ENHS	NO	NO	NO	Dorm	15	2	F	0	5.5	0	10	1	7.5	0	1.5	18	1.5	0
197	Asian	BARNEY	NO	NO	NO	House	75	1	F	0	7.5	2	23	1	1.5	1.5	3.5	19	0	5
198	White	A&S	Non-Contact	NO	NO	On-Campus	225	4	F	0	4.5	2	0	2	3.5	0	7.5	21	0	5
199	Black	CETA	Non-Contact	NO	NO	On-Campus	195	3	M	0	4.5	2	3	1	5.5	0	1.5	20	0	5
200	Black	A&S	NO	NO	NO	House	75	4	F	0.5	5.5	0	30	0	1.5	0	0	20	0	30
201	White	A&S	NO	NO	Face	House	75	4	M	0	7.5	2	3	1	1.5	1.5	3.5	22	0	5
202	White	A&S	No-equipment	NO	NO	House	75	4	M	10	6.5	4	3	1	3.5	4	1.5	21	0	5
203	White	HARTT/HILLYER	Non-Contact	NO	Face	House	135	3	M	4	6.5	1	3	1	1.5	0	1.5	20	0	5
204	White	ENHS	NO	NO	NO	Off-Campus	75	4	F	0	6.5	3	0	1	3.5	0	5.5	21	0	5
205	White	CETA	NO	NO	Non-Face	On-Campus	15	4	M	0	6.5	0	3	1	3.5	0	1.5	21	0	5
206	Asian	CETA	Equipment	NO	Non-Face	Dorm	75	4	M	0	7.5	0	3	0	5.5	0	0	19	0	5
207	Latino	CETA	NO	NO	NO	On-Campus	75	3	M	0	7.5	2	3	2	5.5	0	1.5	19	0	5
208	Black	ENHS	NO	NO	NO	On-Campus	135	4	F	0	7.5	1	0	0	3.5	0	1.5	19	0	0
209	White	CETA	NO	NO	NO	House	75	4	M	0.5	6.5	3	3	3	1.5	0	1.5	21	0	5
210	Asian	ENHS	NO	NO	NO	Off-Campus	135	4	F	0	6.5	1	10	1	1.5	1.5	1.5	22	0	15
211	Asian	CETA	No-equipment	NO	NO	House	135	4	M	0	4.5	0	0	0	1.5	0	1.5	20	0	30
212	White	CETA	NO	NO	NO	House	15	4	M	0	4.5	4	0	1	3.5	0	1.5	20	0	30
213	White	ENHS	NO	Had	Non-Face	Off-Campus	135	4	M	0	5.5	2	30	1	1.5	0	1.5	22	0	30
214	White	CETA	NO	NO	NO	Off-Campus	15	4	M	0	8.5	2	0	2	0	0	3.5	20	0	0
215	Black	A&S	NO	NO	NO	House	75	4	F	0	6.5	2	3	3	0	0	0	22	1.5	15
216	Latino	HARTT/HILLYER	NO	NO	NO	House	15	4	F	0	5.5	3	3	2	1.5	1.5	7.5	18	3.5	30
217	White	ENHS	NO	NO	NO	On-Campus	75	2	F	0	5.5	3	30	2	3.5	4	5.5	21	0	5
218	White	ENHS	NO	NO	NO	Off-Campus	15	4	M	0	5.5	2	10	0	1.5	0	1.5	22	0	0
219	Other	ENHS	NO	Lived	NO	Off-Campus	15	4	F	0	6.5	3	10	1	0	0	0	22	7.5	0
220	White	CETA	NO	NO	NO	House	75	2	M	0	8.5	1	0	1	3.5	0	1.5	18	0	30
221	White	CETA	NO	NO	Non-Face	House	15	4	M	0	5.5	0	3	0	1.5	0	3.5	22	0	15
222	White	ENHS	NO	Lived	NO	Off-Campus	135	0	F	0	7.5	0	10	0	1.5	1.5	1.5	22	0	30
223	White	ENHS	NO	NO	Face	House	15	2	F	0	6.5	1	0	0	3.5	1.5	3.5	22	0	30
224	White	CETA	NO	NO	NO	House	15	2	F	0	5.5	0	3	0	1.5	0	3.5	22	7.5	0
225	Latino	HARTT/HILLYER	NO	NO	NO	House	15	1	M	0	5.5	2	3	0	3.5	0	1.5	19	0	0
226	White	HARTT/HILLYER	NO	NO	NO	Dorm	75	2	M	0	5.5	4	0	0	3.5	1.5	0	18	0	5
227	White	A&S	NO	NO	Non-Face	Off-Campus	75	4	M	10	5.5	4	0	1	1.5	4	1.5	22	0	15
228	White	CETA	NO	NO	Face	Dorm	15	4	M	0	7.5	2	3	0	7.5	0	1.5	18	0	5
229	White	CETA	Non-Contact	NO	NO	Dorm	15	0	M	0	6.5	1	0	0	7.5	0	0	18	0	0
230	Black	CETA	NO	NO	NO	Dorm	75	3	F	0	7.5	2	10	0	7.5	0	0	18	1.5	5
231	Black	CETA	NO	NO	NO	On-Campus	135	4	M	0	5.5	0	3	0	1.5	0	0	19	0	0
232	White	CETA	NO	Had	Non-Face	House	15	2	M	0	7.5	1	0	1	3.5	0	3.5	22	0	30
233	Asian	CETA	No-equipment	NO	NO	House	135	2	M	0	4.5	3	3	1	3.5	0	7.5	19	0	5
234	Black	A&S	NO	Lived	NO	On-Campus	75	4	F	0	6.5	0	10	3	3.5	1.5	0	19	0	0
235	White	ENHS	NO	NO	Non-Face	Off-Campus	15	3	M	0	5.5	0	30	1	3.5	0	1.5	22	0	5
236	Asian	ENHS	NO	NO	NO	On-Campus	75	3	F	0	6.5	1	23	0	3.5	0	7.5	21	0	5
237	Black	ENHS	NO	NO	NO	On-Campus	15	4	F	0	6.5	0	0	1	3.5	0	0	19	0	0
238	Latino	A&S	NO	NO	NO	On-Campus	15	4	M	0	5.5	3	0	0	3.5	0	0	20	1.5	5
239	White	ENHS	NO	NO	NO	House	135	4	M	0	6.5	2	30	0	1.5	0	1.5	22	0	5
240	White	ENHS	NO	NO	NO	Off-Campus	135	4	F	0	5.5	1	30	0	1.5	7.5	5.5	22	0	0
241	White	ENHS	No-equipment	Lived	NO	House	135	3	M	0	6.5	3	30	1	3.5	1.5	1.5	22	0	30
242	White	ENHS	NO	Had	NO	House	135	3	M	0	6.5	2	3	0	3.5	0	1.5	22	0	30
243	White	ENHS	NO	Had	NO	Dorm	75	4	F	0	4.5	1	3	1	7.5	0	0	19	0	15
244	Black	A&S	Non-Contact	NO	NO	On-Campus	15	1	M	10	6.5	1	0	4	5.5	1.5	3.5	21	1.5	15
245	Black	ENHS	NO	NO	NO	Off-Campus	75	3	F	0	6.5	2	10	0	1.5	0	7.5	22	0	5
246	Other	A&S	NO	NO	NO	Off-Campus	135	3	F	0	7.5	2	3	0	1.5	0	1.5	20	0	5
247	White	CETA	NO	NO	NO	On-Campus	75	4	F	0	8.5	3	0	1	5.5	0	5.5	20	0	5
248	White	ENHS	Non-Contact	NO	Face	House	15	4	M	0	6.5	0	0	1	3.5	0	1.5	22	0	30
249	White	ENHS	NO	NO	NO	House	15	4	F	0	7.5	1	3	1	3.5	0	5.5	22	0	30
250	White	CETA	Non-Contact	NO	Non-Face	On-Campus	15	1	M	0	7.5	0	10	0	3.5	0	1.5	21	0	0
251	White	ENHS	Equipment	Had	NO	On-Campus	195	3	F	0	6.5	1	0	1	3.5	0	0	20	0	15
252	White	HARTT/HILLYER	NO	NO	NO	Dorm	15	2	M	0	6.5	0	3	0	7.5	0	0	19	0	0
253	White	HARTT/HILLYER	No-equipment	NO	Non-Face	Dorm	15	3	M	0	8.5	1	0	0	7.5	0	1.5	18	1.5	5
254	White	HARTT/HILLYER	NO	NO	Non-Face	House	15	3	F	4	6.5	1	3	1	3.5	0	1.5	18	0	30
255	Latino	HARTT/HILLYER	NO	NO	Non-Face	House	15	2	F	0	4.5	3	10	1	1.5	0	0	18	0	30

256	Black	HARTT/HILLYER	No-equipment	NO	NO	Dorm	75	3	M	0	6.5	3	3	1	7.5	0	0	18	0	0
257	White	CETA	NO	NO	Non-Face	House	75	1	M	0.5	7.5	2	3	0	0	0	0	22	0	30
258	White	ENHS	Equipment	NO	NO	On-Campus	225	2	F	0	7.5	2	0	1	1.5	0	1.5	20	0	0
259	White	A&S	Equipment	NO	NO	On-Campus	225	4	F	0	6.5	3	3	0	3.5	0	1.5	19	0	15
260	White	ENHS	Equipment	Had	NO	Dorm	225	4	F	0	7.5	3	3	4	3.5	1.5	7.5	18	0	5
261	White	A&S	NO	NO	Non-Face	House	15	4	M	0	5.5	1	0	0	3.5	1.5	1.5	19	0	30
262	Asian	ENHS	Non-Contact	NO	NO	House	15	4	F	0	7.5	1	30	0	3.5	0	7.5	22	0	5
263	White	A&S	NO	NO	NO	Off-Campus	15	4	F	0	6.5	4	3	1	1.5	0	5.5	22	0	30
264	White	CETA	NO	Lived	Non-Face	On-Campus	15	3	M	10	6.5	2	0	1	3.5	0	1.5	19	0	5
265	White	A&S	Non-Contact	NO	Non-Face	House	75	4	F	0	6.5	1	0	0	0	1.5	5.5	21	0	0
266	White	CETA	NO	NO	NO	House	15	4	M	0	7.5	0	0	0	1.5	0	0	22	0	30
267	White	BARNEY	NO	NO	NO	House	75	4	M	0	6.5	2	0	0	0	0	1.5	22	0	0
268	White	BARNEY	NO	NO	NO	On-Campus	135	2	M	0	5.5	1	3	2	5.5	0	1.5	21	0	0
269	White	ENHS	Non-Contact	NO	NO	House	135	4	F	0	7.5	2	3	0	3.5	0	5.5	18	0	30
270	White	ENHS	NO	NO	Face	Dorm	15	4	F	0	7.5	4	0	2	7.5	0	7.5	20	0	5
271	White	CETA	NO	NO	NO	Off-Campus	135	4	F	0	4.5	0	0	1	5.5	0	1.5	20	0	30
272	White	A&S	Non-Contact	NO	NO	House	15	3	M	4	4.5	4	3	1	1.5	0	3.5	18	0	30
273	White	A&S	Non-Contact	NO	NO	Dorm	225	3	M	0	5.5	1	3	1	7.5	0	1.5	19	0	0
274	Latino	A&S	NO	NO	NO	Dorm	15	4	F	4	6.5	0	3	1	7.5	0	5.5	18	5.5	5
275	White	ENHS	NO	NO	NO	Dorm	75	4	F	0	6.5	0	3	2	7.5	0	5.5	18	0	15
276	White	ENHS	NO	NO	NO	Dorm	75	4	F	0	7.5	2	3	2	7.5	0	3.5	18	1.5	5
277	Black	A&S	Non-Contact	NO	NO	Dorm	15	2	F	0	7.5	2	0	2	5.5	4	1.5	18	0	5
278	White	A&S	NO	Had-Lived	Non-Face	Off-Campus	15	3	F	0	7.5	0	23	2	1.5	0	5.5	22	0	30
279	Latino	A&S	NO	NO	NO	Dorm	75	4	F	10	6.5	2	10	3	5.5	0	5.5	18	0	15
280	White	A&S	No-equipment	NO	Face	On-Campus	135	4	F	0	7.5	2	23	0	5.5	0	5.5	20	0	5
281	Other	A&S	NO	NO	NO	Dorm	15	4	F	0	6.5	2	0	1	7.5	0	0	18	1.5	0
282	Black	A&S	NO	NO	Face	Dorm	15	4	F	0	5.5	0	0	0	3.5	0	0	18	0	0
283	White	A&S	NO	NO	NO	House	15	3	F	0	5.5	0	0	0	0	0	7.5	19	0	0
284	Other	A&S	NO	NO	NO	Dorm	75	1	F	0	6.5	2	0	1	5.5	1.5	0	18	1.5	5
285	Latino	A&S	NO	NO	Face	Dorm	75	3	F	0	6.5	2	0	0	7.5	0	1.5	19	0	5
286	Black	ENHS	NO	NO	NO	Dorm	15	4	F	0	6.5	4	3	1	7.5	0	3.5	21	0	0
287	White	ENHS	NO	NO	Non-Face	On-Campus	15	4	F	0	7.5	2	0	0	3.5	1.5	1.5	21	0	5
288	White	A&S	NO	NO	NO	House	15	2	M	0	8.5	2	3	1	3.5	0	1.5	21	0	30
289	Black	ENHS	NO	NO	NO	On-Campus	15	4	M	0	6.5	2	30	0	1.5	0	1.5	21	0	15
290	White	ENHS	NO	NO	NO	House	75	3	F	0	6.5	2	0	0	1.5	0	1.5	19	0	30
291	Black	ENHS	NO	NO	NO	House	75	4	F	0	7.5	2	0	1	7.5	0	0	19	1.5	0
292	White	ENHS	NO	Had	NO	Dorm	15	1	F	1	5.5	2	30	1	5.5	0	3.5	18	0	5
293	White	ENHS	NO	NO	NO	Dorm	75	2	F	0	6.5	3	0	1	5.5	0	5.5	18	1.5	5
294	White	ENHS	Non-Contact	Had	NO	Dorm	75	3	F	0	6.5	1	3	2	7.5	0	5.5	18	1.5	15
295	White	ENHS	NO	Lived	NO	Dorm	15	4	F	0	6.5	0	0	2	7.5	0	3.5	18	0	5
296	Black	ENHS	NO	NO	NO	Dorm	15	4	F	0	7.5	2	0	1	7.5	0	0	18	1.5	5
297	Black	A&S	NO	NO	NO	Dorm	75	4	F	0	6.5	2	3	0	7.5	1.5	3.5	18	1.5	5
298	White	ENHS	NO	NO	NO	Dorm	15	3	F	0	4.5	4	3	0	5.5	1.5	3.5	19	1.5	0
299	White	ENHS	Non-Contact	NO	NO	Dorm	75	2	M	4	6.5	2	30	1	1.5	0	1.5	18	0	0
300	White	ENHS	NO	NO	NO	Dorm	15	4	F	0	6.5	2	3	2	7.5	0	1.5	18	1.5	5
301	White	ENHS	Equipment	NO	NO	On-Campus	135	4	F	0	6.5	2	3	1	3.5	0	1.5	20	0	5
302	Black	A&S	NO	NO	NO	House	15	3	F	0	5.5	3	3	1	7.5	0	0	22	0	0
303	White	A&S	NO	Had	NO	House	75	2	F	0	7.5	2	3	4	3.5	0	3.5	20	0	30
304	White	A&S	NO	NO	NO	House	135	4	F	10	5.5	0	0	0	3.5	0	3.5	21	0	5
305	Asian	CETA	Equipment	NO	Non-Face	House	75	1	M	0	5.5	1	3	1	1.5	0	0	22	0	5
306	Latino	CETA	No-equipment	NO	NO	On-Campus	75	3	F	0	6.5	2	3	1	3.5	1.5	1.5	21	0	5
307	Latino	A&S	NO	Lived	NO	House	15	4	F	0	6.5	2	30	0	5.5	7.5	0	19	0	30
308	White	ENHS	NO	NO	Non-Face	Dorm	15	4	F	0	6.5	2	3	0	7.5	0	0	18	0	5
309	Black	A&S	NO	NO	NO	Off-Campus	15	3	F	0	6.5	0	3	0	1.5	0	0	20	7.5	0
310	White	A&S	Equipment	NO	NO	On-Campus	135	3	F	0	6.5	2	0	2	1.5	1.5	3.5	20	0	5
311	White	CETA	NO	NO	Non-Face	Off-Campus	15	2	O	10	7.5	4	3	0	3.5	1.5	1.5	22	0	0
312	White	BARNEY	Non-Contact	NO	NO	House	15	3	M	0	6.5	2	3	1	3.5	0	3.5	19	0	5
313	Latino	HARTT/HILLYER	NO	NO	NO	On-Campus	75	4	F	0	5.5	0	10	0	3.5	0	3.5	21	0	5
314	White	A&S	NO	NO	NO	On-Campus	15	4	F	0	8.5	0	3	2	5.5	1.5	0	20	0	5
315	White	ENHS	No-equipment	NO	NO	On-Campus	225	4	F	0	4.5	2	3	1	1.5	1.5	1.5	20	0	0
316	Latino	BARNEY	Non-Contact	NO	Face	On-Campus	135	3	M	0	5.5	3	0	0	3.5	0	1.5	19	0	5
317	White	ENHS	NO	Had	Face	House	15	0	O	0	8.5	0	3	0	5.5	0	3.5	18	5.5	30
318	White	CETA	Non-Contact	NO	Non-Face	Dorm	135	4	M	4	4.5	0	3	2	5.5	0	1.5	19	0	5
319	White	HARTT/HILLYER	NO	NO	Face	On-Campus	75	0	M	0	6.5	2	3	0	3.5	0	3.5	19	1.5	5
320	White	A&S	NO	NO	NO	Dorm	15	2	M	0	6.5	4	10	2	7.5	0	1.5	19	0	5
321	White	HARTT/HILLYER	NO	NO	Non-Face	Dorm	15	2	M	0	7.5	1	3	1	1.5	0	0	19	0	0
322	White	ENHS	NO	NO	Face	Dorm	75	4	F	0	7.5	2	30	0	3.5	0	0	19	0	15
323	White	HARTT/HILLYER	NO	NO	NO	House	15	0	F	0	7.5	1	0	0	0	0	1.5	19	1.5	30
324	White	CETA	Non-Contact	NO	Non-Face	Dorm	15	4	M	0	7.5	2	0	0	7.5	0	1.5	21	1.5	5
325	White	A&S	NO	NO	NO	Off-Campus	135	4	F	0	7.5	2	3	2	1.5	0	1.5	22	0	25
326	Latino	A&S	NO	NO	NO	On-Campus	75	4	F	0	7.5	2	3	1	3.5	0	5.5	20	0	0
327	White	A&S	NO	NO	NO	On-Campus	75	4	M	0.5	7.5	2	3	1	3.5	0	1.5	21	0	25
328	White	ENHS	NO	NO	NO	House	15	4	F	0	8.5	0	0	1	3.5	4	5.5	21	0	30
329	White	ENHS	NO	NO	NO	On-Campus	75	4	F	0	7.5	1	3	2	3.5	4	1.5	21	0	0
330	Asian	ENHS	NO	NO	NO	On-Campus	75	3	F	0	6.5	1	3	1	3.5	0	1.5	19	3.5	5
331	Black	A&S	NO	NO	NO	House	15	4	F	0	4.5	2	3	1	1.5	0	0	22	0	0
332	Latino	A&S	NO	NO	NO	House	15	3	F	0	7.5	0	3	1	3.5	0	0	18	3.5	5
333	White	A&S	NO	NO	Face	On-Campus	15	4	M	0	8.5	1	3	2	3.5	0	1.5	20	0	5
334	White	A&S	NO	NO	NO	Off-Campus	135	2	M	0	6.5	0	0	2	7.5	0	3.5	19	0	30
335	White	A&S	NO	NO	NO	Dorm	15	2	M	0	6.5	4	0	2	1.5	0	0	21	0	0
336	Black	BARNEY	NO	NO	NO	Dorm	15	4	F	0	7.5	2	0	0	3.5	0	0	21	0	0
337	White	ENHS	NO	NO	NO	Dorm	15	2	M	0	6.5	0	3	2	7.5	0	1.5	19	0	5
338	Asian	ENHS	NO	NO	Face	Off-Campus	75	4	F	0	6.5	2	3	2	7.5	0	1.5	22	0	5
339	White	A&S	NO	Had-Lived	NO	House	195	4	F	0	6.5	0	3	2	1.5	1.5	0	22	0	5
340	White	ENHS	NO	NO	NO	Off-Campus	15	2	M	0	6.5	0	0	0	0	0	0	22	0	5
341	Black	ENHS	NO	NO	NO	Dorm	15	2	M	0	7.5	3	0	0	3.5	0	0	20	0	5
342	White	BARNEY	NO	NO	NO	House	15	4	M	0	6.5	2	3	0	3.5	0	1.5	18	0	5
343	Black	ENHS	NO	NO	Non-Face	On-Campus	75	3	M	10	4.5	3	3	0	3.5	0	0	19	0	0
344	White	A&S	NO	NO	NO	Dorm	15	2	F	0	8.5	1	0	2	7.5	0	0	20	0	0
345	Black	HARTT/HILLYER	NO	NO	NO	Dorm	15	4	F	0	8.5	3	3	2	7.5	0	0	18	1.5	5
346	Black	HARTT/HILLYER	NO	NO	NO	Dorm	15	4	F	0	4.5	4	3	2	7.5	0	1.5	18	0	15
347	Black	HARTT/HILLYER	NO	NO	NO	House	195	4	M	0	6.5	2	10	2	1.5	0	1.5	18	7.5	0
348	White	A&S	NO	NO	NO	On-Campus	15	4	F	4	4.5	3	3	0	1.5	1.5	5.5	20	0	30
349	White	HARTT/HILLYER	Non-Contact	NO	Non-Face	House	225	4	M	0	6.5	0	3	1	3.5	0	1.5	22	0	30
350	White	BARNEY	NO	NO	NO	Off-Campus	15	4	M	10	7.5	3	3	2	0	0	1.5	20	0	5
351	White	CETA	No-equipment	NO	Non-Face	On-Campus	75	4	M	0	5.5	2	3	2	3.5	0	1.5	21	0	0
352	White	HARTT/HILLYER	NO	NO	NO	On-Campus	15	4	F	4	8.5	3	3	1	3.5	0	3.5	19	0	5
353	White	HARTT/HILLYER	NO	NO	NO	On-Campus	15	4	F	4	7.5	3	3	0	3.5	4	5.5	18	0	5
354	White	A&S	NO	NO	NO	On-Campus	15	4	M	0	7.5	2	0	0	3.5	0	1.5	20	0	5
355	White	A&S	NO	NO	NO	On-Campus	15	4	F	0	6.5	3	0	0	3.5	4	5.5	20	0	5
356	Black	CETA	Non-Contact	NO	NO	Dorm	195	1	M	0	4.5	2	3	0	5.5	0	1.5	22	0	0
357	Black	BARNEY	Non-Contact	NO	NO	On-Campus	135	2	M	0	5.5	1	3	0	3.5	0	0	19	1.5	5
358	Black	A&S	NO	NO	NO	Dorm	15	4	F	0	4.5	1	0	0	3.5	0	3.5	20	0	0
359	White	ENHS	NO	NO	Non-Face	House	75	3	M	0	6.5	0	0	0	3.5	0	1.5	21	0	30
360	Latino	A&S	No-equipment	NO	NO	House	15	4	M	0	6.5	2	3	0	3.5	0	1.5	20	0	30
361	Black	A&S	NO	NO	NO	House	75	3	M	0	7.5	2	10	1	1.5	0	0	20	0	30
362	White	BARNEY	NO	NO	NO	House	75	3	M	10	8.5	1	3	0	3.5	0	0	18	0	30
363	Latino	ENHS	NO	NO	NO	Dorm	135	3	F	0	6.5	2	10	2	7.5	1.5	7.5	21	0	0
364	White	A&S	NO	NO	NO	House	15	3	M	0	7.5	4	3	0	1.5	0	1.5	19	0	0
365	White	A&S	NO	NO	NO	Dorm	15	2	M	0	5.5	4	30	4	5.5	0	0	20	0	0
366	Black	A&S	NO	NO	NO	On-Campus	15	3	F	0	8.5	4	3	2	3.5	0	0	19	0	30
367	White	HARTT/HILLYER	NO	NO	Non-Face	Off-Campus	15	3	M	0	7.5	2	3	0	1.5	0	1.5	20	1.5	5
368	Other	A&S	NO	NO	NO	On-Campus	15	4	F	0	5.5	2	0	2	0	0	1.5	22	0	0
369	White	A&S	NO	NO	NO	On-Campus	15	4	M	4	7.5	0	23	3	3.5	0	3.5	21	0	25
370	Asian	BARNEY	NO	NO	NO	Dorm	75	4	M	0	4.5	1	3	3	7.5	0	0	18	0	15
371	Black	HARTT/HILLYER	Shared	NO	Non-Face	Dorm	135	1	M	0	5.5	2	23	2	1.5	0	0	18	0	5
372	White	BARNEY	Non-Contact	NO	NO	Dorm	15	3	M	4	7.5	0	0	0	3.5	1.5	1.5	20	0	5
373	Other	A&S	NO	NO	NO	Dorm	195	3	F	0	6.5	1	0	1	1.5	1.5	5.5	21	0	5
374	White	HARTT/HILLYER	Non-Contact	NO	NO	On-Campus	15	4	F	0	7.5	1	3	1	1.5	0	1.5	20	0	5
375	Black	A&S	NO	NO	NO	Dorm	15	3	F	0	4.5	0	3	0	7.5	0	0	19	0	0
376	Black	A&S	NO	NO	Face	Dorm	135	1	F	0	6.5	2	3	2	5.5	0	1.5	18	0	5
377	White	A&S	NO	NO	Face	On-Campus	75	4	M	0	5.5	2	3	0	5.5	0	0	22	3.5	5
378	White	ENHS	NO	NO	NO	On-Campus	75	4	F	0.5	7.5	2	10	4	3.5	0	3.5	19	0	5
379	White	ENHS	Non-Contact	NO	NO	Off-Campus	135	4	M	0	5.5	2	3	1	1.5	1.5	3.5	22	0	5
380	White	ENHS	Equipment	Lived	NO	Off-Campus	15	4	M	0	6.5	1	30	1	0	0	1.5	22	0	5
381	Latino	ENHS	No-equipment	NO	NO	Off-Campus	135	4	F	0	6.5	0	23	1	0	1.5	0	22	0	30
382	Latino	ENHS	NO	NO	NO	Off-Campus	75	4	F	0	6.5	0	30	0	1.5	0	0	22	0	0
383	Latino	A&S	NO	Lived	NO	On-Campus	15	3	F	0	7.5	0	3	1	3.5	7.5	7.5	21	0	15
384	Latino	HARTT/HILLYER	Equipment	NO	Non-Face	Dorm	135	3	F	0	5.5	3	3	1	7.5	0	0	18	3.5	5

385	White	BARNEY	NO	NO	Face	Off-Campus	15	0	M	0	7.5	0	30	2	1.5	0	1.5	22	0	30
386	White	ENHS	NO	NO	NO	Dorm	135	4	F	0	7.5	2	3	4	5.5	0	1.5	19	1.5	5
387	White	ENHS	NO	NO	NO	Dorm	135	4	M	0	6.5	2	3	2	3.5	0	1.5	20	0	5
388	Black	A&S	NO	NO	NO	On-Campus	15	2	F	0	6.5	3	3	0	3.5	0	7.5	19	1.5	0
389	Latino	A&S	NO	NO	NO	Dorm	15	4	F	0	6.5	3	3	1	5.5	1.5	1.5	20	0	5
390	White	ENHS	No-equipment	NO	NO	On-Campus	225	2	F	0	7.5	2	3	4	5.5	0	1.5	19	0	5
391	White	ENHS	Non-Contact	NO	NO	House	15	3	M	10	6.5	2	0	0	3.5	0	1.5	20	0	30
392	Other	A&S	NO	NO	NO	Dorm	15	4	F	1	5.5	3	3	1	5.5	1.5	1.5	19	0	5
393	Latino	CETA	NO	Had	NO	Dorm	15	4	F	0	7.5	3	3	2	7.5	0	3.5	19	0	0
394	Black	A&S	NO	NO	NO	On-Campus	15	3	F	0	7.5	1	3	0	3.5	1.5	0	19	0	5
395	Black	ENHS	NO	NO	NO	On-Campus	135	4	M	0	5.5	3	0	1	3.5	1.5	0	19	1.5	30
396	Black	BARNEY	No-equipment	NO	NO	On-Campus	195	4	M	0	6.5	3	3	0	3.5	0	0	18	0	0
397	Latino	A&S	NO	NO	NO	Off-Campus	15	3	F	0	7.5	2	0	1	1.5	1.5	7.5	22	0	0
398	White	HARTT/HILLYER	NO	NO	NO	On-Campus	15	2	M	0	6.5	1	3	0	3.5	0	1.5	19	1.5	0
399	Black	HARTT/HILLYER	Non-Contact	NO	Non-Face	Dorm	15	2	F	0	7.5	0	3	2	3.5	0	0	18	0	25
400	Black	A&S	NO	NO	NO	On-Campus	75	3	M	1	6.5	4	3	1	3.5	1.5	3.5	20	0	5
401	White	A&S	NO	NO	NO	On-Campus	15	4	F	0	7.5	0	23	3	3.5	1.5	5.5	20	0	25
402	Black	A&S	Non-Contact	NO	NO	Dorm	225	2	M	0	5.5	1	3	0	5.5	0	0	18	0	5
403	Asian	BARNEY	No-equipment	Lived	NO	Dorm	225	4	M	0	5.5	3	3	2	7.5	0	3.5	18	1.5	0
404	White	HARTT/HILLYER	Non-Contact	NO	Face	Dorm	225	1	M	0	6.5	1	3	0	7.5	0	1.5	18	1.5	5
405	Black	BARNEY	No-equipment	NO	NO	Dorm	135	1	M	0	7.5	2	3	0	7.5	0	0	18	0	0
406	Black	CETA	Non-Contact	NO	NO	On-Campus	135	1	M	0	4.5	1	3	0	5.5	0	1.5	20	0	5
407	White	A&S	Non-Contact	Lived	NO	Dorm	75	0	M	0	4.5	3	3	1	3.5	0	1.5	18	0	5
408	Black	BARNEY	Non-Contact	NO	NO	Dorm	135	4	M	0	5.5	4	3	2	3.5	7.5	0	19	0	5
409	White	BARNEY	Non-Contact	NO	NO	On-Campus	225	3	F	0	6.5	4	0	2	3.5	0	7.5	20	0	15
410	Latino	A&S	Non-Contact	NO	NO	On-Campus	135	4	M	0	5.5	3	3	1	3.5	0	1.5	19	0	0
411	Black	ENHS	Non-Contact	NO	Face	On-Campus	75	2	M	0	4.5	2	3	1	3.5	0	0	19	0	0
412	Black	CETA	Non-Contact	NO	NO	On-Campus	195	4	M	4	4.5	3	3	1	3.5	0	1.5	21	0	5
413	Latino	A&S	NO	NO	Non-Face	House	75	4	M	0	6.5	2	3	2	1.5	0	1.5	22	7.5	5
414	White	CETA	No-equipment	NO	NO	On-Campus	75	3	M	0	5.5	4	3	2	3.5	1.5	1.5	21	0	5
415	White	HARTT/HILLYER	No-equipment	NO	Face	House	135	4	M	0	6.5	0	3	1	3.5	0	1.5	20	0	30
416	Latino	A&S	NO	NO	NO	Off-Campus	75	4	M	4	6.5	0	0	0	0	10	1.5	21	0	0
417	White	ENHS	Non-Contact	NO	NO	On-Campus	225	4	M	0	7.5	0	3	1	3.5	0	1.5	19	0	0
418	Black	BARNEY	Shared	NO	NO	Dorm	135	4	F	0	7.5	4	3	1	5.5	0	0	19	0	0
419	White	BARNEY	No-equipment	NO	NO	On-Campus	135	4	M	0	6.5	1	3	2	3.5	1.5	1.5	20	0	5
420	White	HARTT/HILLYER	NO	NO	Non-Face	Off-Campus	135	1	M	0	5.5	1	3	2	1.5	0	0	20	0	0
421	White	A&S	Shared	NO	NO	On-Campus	195	4	F	0	6.5	1	10	2	3.5	1.5	3.5	20	3.5	5
422	Other	A&S	No-equipment	NO	NO	On-Campus	225	4	M	0	7.5	1	3	3	7.5	0	1.5	19	0	0
423	White	ENHS	Equipment	NO	NO	On-Campus	195	4	F	0	6.5	2	10	2	1.5	0	1.5	21	0	5
424	White	BARNEY	Equipment	NO	NO	On-Campus	195	2	F	0	7.5	3	3	1	3.5	0	1.5	19	0	5
425	White	ENHS	Shared	NO	NO	Dorm	135	4	M	0	5.5	1	3	0	7.5	0	1.5	18	0	5
426	White	A&S	Non-Contact	NO	NO	On-Campus	195	2	F	0	7.5	3	3	1	3.5	1.5	3.5	21	0	0
427	White	ENHS	Equipment	NO	NO	On-Campus	195	4	F	4	6.5	2	3	2	3.5	1.5	1.5	19	0	5
428	White	ENHS	No-equipment	NO	NO	Dorm	195	4	F	0	5.5	1	3	2	7.5	0	0	19	1.5	5
429	White	ENHS	Non-Contact	NO	NO	Dorm	225	4	M	0	7.5	2	3	0	7.5	0	0	18	0	0
430	Latino	CETA	NO	NO	NO	On-Campus	135	4	M	0	4.5	0	3	0	1.5	0	1.5	19	0	0
431	White	BARNEY	No-equipment	NO	NO	On-Campus	135	4	F	0	7.5	1	0	0	3.5	1.5	5.5	20	0	0
432	Latino	CETA	Non-Contact	NO	NO	Dorm	135	1	M	0	5.5	3	3	2	7.5	0	1.5	18	0	25
433	White	A&S	NO	NO	Non-Face	Dorm	135	3	F	0	6.5	0	0	3	7.5	1.5	1.5	19	0	25
434	White	ENHS	No-equipment	NO	NO	Dorm	195	2	F	0	5.5	2	3	1	7.5	0	3.5	18	0	15
435	White	ENHS	Non-Contact	NO	NO	Dorm	225	3	F	0	7.5	2	3	1	7.5	0	0	18	3.5	15
436	White	ENHS	Non-Contact	NO	Face	Dorm	225	3	F	0	5.5	1	0	1	7.5	0	1.5	18	0	5
437	White	ENHS	Non-Contact	NO	NO	On-Campus	225	3	F	0	5.5	2	3	1	3.5	0	1.5	19	0	5
438	White	ENHS	Non-Contact	NO	NO	Dorm	195	4	F	10	6.5	3	0	2	3.5	1.5	1.5	19	0	15
439	Black	A&S	Non-Contact	NO	NO	Dorm	195	3	F	0	5.5	4	3	2	7.5	1.5	1.5	18	0	0
440	White	HARTT/HILLYER	NO	NO	Face	Off-Campus	135	4	F	0	5.5	1	3	1	1.5	0	5.5	21	0	0
441	White	ENHS	NO	NO	NO	Dorm	195	2	M	0	5.5	3	10	1	7.5	0	1.5	18	0	15
442	White	HARTT/HILLYER	No-equipment	NO	Face	House	135	3	M	0	5.5	1	3	0	3.5	0	1.5	20	1.5	5
443	White	CETA	NO	NO	NO	On-Campus	135	3	M	0	6.5	3	3	1	5.5	0	0	18	0	5
444	White	A&S	Equipment	NO	NO	On-Campus	225	3	F	0	4.5	3	10	3	3.5	4	3.5	21	0	30
445	White	A&S	Shared	NO	Non-Face	On-Campus	225	3	F	0	5.5	4	3	1	5.5	4	3.5	21	0	30
446	White	HARTT/HILLYER	NO	NO	Non-Face	House	195	4	M	10	6.5	2	0	1	1.5	1.5	1.5	21	0	0
447	White	BARNEY	NO	NO	NO	Off-Campus	195	4	M	0	5.5	2	3	4	0	1.5	3.5	22	0	5
448	White	HARTT/HILLYER	Non-Contact	NO	Face	Off-Campus	75	1	M	0	6.5	1	0	1	0	0	1.5	22	0	30
449	White	BARNEY	No-equipment	NO	NO	Off-Campus	135	4	M	1	7.5	2	3	1	1.5	0	0	21	0	5
450	Latino	BARNEY	No-equipment	NO	NO	Off-Campus	135	2	M	0	8.5	2	0	0	1.5	0	0	19	0	5
451	White	A&S	Non-Contact	NO	NO	Dorm	225	1	M	0	8.5	2	3	0	7.5	0	1.5	19	0	0
452	Latino	CETA	NO	NO	NO	On-Campus	75	4	M	0	5.5	2	0	1	1.5	0	0	19	0	5
453	Black	CETA	Equipment	NO	NO	On-Campus	195	4	M	0	5.5	1	3	2	3.5	0	0	18	0	0
454	Latino	CETA	NO	NO	NO	On-Campus	135	2	M	0	5.5	2	0	0	3.5	0	0	19	0	0
455	White	ENHS	NO	NO	NO	On-Campus	135	4	M	0	6.5	1	30	1	3.5	0	3.5	21	0	5
456	White	BARNEY	NO	NO	NO	On-Campus	135	4	M	0	6.5	0	3	0	3.5	0	1.5	20	0	5
457	White	BARNEY	Non-Contact	NO	NO	On-Campus	225	4	M	0	5.5	1	0	0	1.5	0	0	21	0	0
458	White	BARNEY	No-equipment	NO	NO	Dorm	195	2	M	0	6.5	1	3	2	7.5	0	0	18	0	5
459	White	ENHS	Non-Contact	NO	NO	Dorm	225	2	M	0	7.5	2	3	1	7.5	0	3.5	18	0	15
460	White	ENHS	Non-Contact	NO	NO	Dorm	195	3	M	0	7.5	0	3	1	7.5	0	1.5	19	0	0
461	White	A&S	NO	NO	Non-Face	Dorm	75	2	F	0	6.5	2	0	3	7.5	1.5	0	18	0	5
462	White	HARTT/HILLYER	NO	NO	Face	Off-Campus	15	1	F	0	7.5	1	3	0	3.5	0	1.5	20	3.5	5
463	White	CETA	NO	NO	NO	Dorm	15	2	F	4	4.5	1	3	1	5.5	4	1.5	21	0	0
464	White	CETA	NO	NO	NO	Dorm	15	2	F	0	4.5	1	0	0	7.5	0	1.5	18	0	0
465	White	ENHS	No-equipment	Lived	Face	Dorm	225	2	M	0	5.5	0	3	0	7.5	0	1.5	18	0	5
466	White	A&S	No-equipment	NO	NO	Dorm	225	1	M	0	6.5	2	0	0	7.5	0	1.5	19	0	5
467	White	A&S	Non-Contact	NO	NO	On-Campus	225	3	M	0	7.5	3	3	1	3.5	0	1.5	20	0	5
468	White	A&S	Equipment	NO	NO	On-Campus	195	4	F	0	7.5	4	3	1	3.5	4	7.5	21	0	5
469	White	ENHS	Equipment	NO	NO	Dorm	135	1	F	0	5.5	2	3	2	7.5	0	3.5	18	1.5	5
470	White	ENHS	Equipment	NO	Face	Dorm	135	1	F	0	6.5	4	0	2	5.5	0	0	18	3.5	0
471	White	A&S	NO	NO	NO	Dorm	75	4	F	0	7.5	2	3	0	7.5	0	1.5	18	1.5	5
472	Black	A&S	NO	NO	NO	House	75	1	F	0	7.5	2	0	0	0	1.5	1.5	21	0	0
473	White	HARTT/HILLYER	NO	NO	NO	Dorm	75	3	M	0	6.5	0	3	0	3.5	0	1.5	18	0	5
474	White	HARTT/HILLYER	NO	NO	Face	On-Campus	15	2	M	1	6.5	2	0	0	1.5	0	0	19	0	5
475	White	HARTT/HILLYER	NO	NO	Face	Dorm	75	3	M	10	6.5	2	0	0	5.5	0	1.5	20	0	5
476	White	HARTT/HILLYER	NO	NO	Non-Face	On-Campus	135	3	F	0	8.5	1	3	3	3.5	0	1.5	19	0	5
477	White	A&S	NO	NO	NO	House	15	4	F	0	7.5	0	3	1	1.5	7.5	3.5	22	0	30
478	White	A&S	NO	NO	NO	House	15	2	M	0	6.5	1	0	0	0	0	3.5	22	0	0
479	White	ENHS	Non-Contact	NO	NO	Dorm	75	4	F	0	5.5	0	3	0	5.5	7.5	5.5	18	0	25
480	White	ENHS	NO	Lived	NO	Dorm	75	1	F	0	7.5	2	3	4	7.5	1.5	3.5	18	0	5
481	White	BARNEY	NO	NO	NO	Dorm	15	3	M	0	6.5	0	3	0	7.5	0	1.5	18	0	30
482	White	HARTT/HILLYER	NO	NO	NO	Dorm	15	4	M	0	6.5	1	0	0	7.5	0	0	19	1.5	0
483	Latino	A&S	NO	NO	NO	House	15	3	F	0	6.5	0	3	0	3.5	0	0	18	0	5
484	Black	CETA	No-equipment	NO	NO	Off-Campus	75	4	M	0	5.5	0	0	1	1.5	0	0	18	0	0
485	White	A&S	NO	Had	NO	Off-Campus	75	2	F	0	6.5	2	3	2	1.5	1.5	1.5	21	0	30
486	White	CETA	NO	NO	Non-Face	On-Campus	75	4	M	0.5	8.5	2	30	1	5.5	0	0	19	0	0
487	Black	A&S	NO	NO	Non-Face	Dorm	135	4	F	0	8.5	3	3	0	7.5	0	0	18	1.5	5
488	Asian	A&S	NO	NO	NO	Dorm	135	3	M	0	6.5	3	0	0	7.5	0	0	18	0	0
489	Black	ENHS	NO	NO	NO	On-Campus	15	4	F	0	6.5	2	0	1	3.5	0	0	19	0	0
490	Black	BARNEY	NO	NO	NO	Dorm	15	3	F	0	7.5	4	0	0	3.5	0	0	19	3.5	0
491	White	BARNEY	NO	NO	Non-Face	Dorm	15	0	M	0	6.5	0	0	0	7.5	0	1.5	18	0	5
492	Other	BARNEY	Equipment	NO	NO	On-Campus	195	2	M	10	5.5	3	3	1	3.5	0	0	20	0	5
493	White	BARNEY	Equipment	NO	NO	House	225	3	M	0	6.5	2	0	0	5.5	0	1.5	20	0	0
494	White	BARNEY	NO	NO	Face	Dorm	75	4	F	0	7.5	2	3	0	7.5	0	1.5	18	0	15
495	White	A&S	Shared	NO	NO	Dorm	195	2	F	0	6.5	2	0	0	5.5	0	1.5	18	1.5	0
496	White	BARNEY	Non-Contact	NO	NO	On-Campus	225	4	F	0	7.5	3	3	2	1.5	0	5.5	21	0	5
497	White	BARNEY	NO	NO	NO	On-Campus	15	3	M	10	7.5	0	0	0	3.5	0	0	21	0	0
498	White	BARNEY	NO	NO	NO	Off-Campus	75	4	F	0	7.5	3	3	3	0	0	5.5	22	0	25
499	Black	BARNEY	Shared	NO	Non-Face	On-Campus	135	1	M	0	7.5	2	0	0	1.5	0	0	21	0	0
500	Black	BARNEY	NO	NO	Non-Face	Dorm	15	2	M	0	6.5	2	0	0	7.5	0	0	21	0	5

Species	Penicillin_Resistance	Methicillin_Resistance	Trimethoprim_Resistance	Tetracycline_Resistance
NONE	NO	NO	NO	NO
NONE	NO	NO	NO	NO
NONE	NO	NO	NO	NO
NONE	NO	NO	NO	NO
NONE	NO	NO	NO	NO
S. haemolyticus	NO	NO	NO	NO
NONE	NO	NO	NO	NO
NONE	NO	NO	NO	NO
NONE	NO	NO	NO	NO
NONE	NO	NO	NO	NO
NONE	NO	NO	NO	NO
S. aureus	YES	NO	NO	NO
NONE	NO	NO	NO	NO
NONE	NO	NO	NO	NO
NONE	NO	NO	NO	NO
NONE	NO	NO	NO	NO
S. aureus	YES	NO	NO	NO
S. aureus	YES	NO	NO	NO
NONE	NO	NO	NO	NO
NONE	NO	NO	NO	NO
S. aureus	YES	NO	NO	NO
NONE	NO	NO	NO	NO
NONE	NO	NO	NO	NO
NONE	NO	NO	NO	NO
NONE	NO	NO	NO	NO
S. aureus	NO	NO	NO	NO
NONE	NO	NO	NO	NO
NONE	NO	NO	NO	NO
NONE	NO	NO	NO	NO
S. aureus	NO	NO	NO	NO
NONE	NO	NO	NO	NO
S. aureus	YES	NO	NO	NO
NONE	NO	NO	NO	NO
NONE	NO	NO	NO	NO
S. warneri	YES	NO	NO	NO
NONE	NO	NO	NO	NO
NONE	NO	NO	NO	NO
NONE	NO	NO	NO	NO
S. capitis	YES	NO	NO	NO
NONE	NO	NO	NO	NO
NONE	NO	NO	NO	NO
NONE	NO	NO	NO	NO
S. haemolyticus	NO	NO	NO	NO
NONE	NO	NO	NO	NO
NONE	NO	NO	NO	NO
NONE	NO	NO	NO	NO
NONE	NO	NO	NO	NO
NONE	NO	NO	NO	NO
S. haemolyticus	NO	NO	NO	NO
NONE	NO	NO	NO	NO
S. aureus	YES	YES	NO	NO
S. aureus	YES	NO	NO	NO
NONE	NO	NO	NO	NO
NONE	NO	NO	NO	NO
NONE	NO	NO	NO	NO
NONE	NO	NO	NO	NO
NONE	NO	NO	NO	NO
NONE	NO	NO	NO	NO
S. aureus	YES	NO	NO	YES
NONE	NO	NO	NO	NO
S. aureus	NO	NO	NO	NO
NONE	NO	NO	NO	NO
NONE	NO	NO	NO	NO
NONE	NO	NO	NO	NO
NONE	NO	NO	NO	NO
NONE	NO	NO	NO	NO
NONE	NO	NO	NO	NO
S. aureus	YES	NO	NO	NO
S. aureus	NO	NO	NO	NO
NONE	NO	NO	NO	NO
NONE	NO	NO	NO	NO
NONE	NO	NO	NO	NO
NONE	NO	NO	NO	NO
NONE	NO	NO	NO	NO
NONE	NO	NO	NO	NO
NONE	NO	NO	NO	NO
S. aureus	NO	NO	NO	YES
NONE	NO	NO	NO	NO
S. saprophyticus	NO	NO	YES	YES
S. aureus	YES	NO	NO	NO
NONE	NO	NO	NO	NO
NONE	NO	NO	NO	NO
NONE	NO	NO	NO	NO
NONE	NO	NO	NO	NO
NONE	NO	NO	NO	NO
NONE	NO	NO	NO	NO
S. aureus	NO	NO	NO	NO
NONE	NO	NO	NO	NO
NONE	NO	NO	NO	NO
S. xylosus	NO	NO	NO	NO
NONE	NO	NO	NO	NO
S. cohnii	NO	NO	NO	NO
NONE	NO	NO	NO	NO
S. aureus	YES	NO	NO	NO
S. aureus	NO	YES	YES	NO
NONE	NO	NO	NO	NO
S. aureus	NO	NO	NO	NO
NONE	NO	NO	NO	NO
NONE	NO	NO	NO	NO
NONE	NO	NO	NO	NO
S. aureus	NO	NO	NO	NO
NONE	NO	NO	NO	NO
NONE	NO	NO	NO	NO
NONE	NO	NO	NO	NO
NONE	NO	NO	NO	NO
NONE	NO	NO	NO	NO
NONE	NO	NO	NO	NO
NONE	NO	NO	NO	NO
NONE	NO	NO	NO	NO
NONE	NO	NO	NO	NO
NONE	NO	NO	NO	NO
NONE	NO	NO	NO	NO
NONE	NO	NO	NO	NO
S. aureus	NO	NO	NO	NO
S. saprophyticus	NO	NO	NO	NO
NONE	NO	NO	NO	NO
NONE	NO	NO	NO	NO
NONE	NO	NO	NO	NO
NONE	NO	NO	NO	NO
S. aureus	YES	NO	NO	NO
NONE	NO	NO	NO	NO
S. aureus	YES	NO	NO	NO
NONE	NO	NO	NO	NO
NONE	NO	NO	NO	NO
NONE	NO	NO	NO	NO
NONE	NO	NO	NO	NO

S. aureus	NO	NO	NO	NO
S. aureus	YES	NO	NO	NO
NONE	NO	NO	NO	NO
NONE	NO	NO	NO	NO
S. warneri	YES	NO	NO	NO
S. aureus	YES	NO	NO	NO
NONE	NO	NO	NO	NO
NONE	NO	NO	NO	NO
NONE	NO	NO	NO	NO
NONE	NO	NO	NO	NO
NONE	NO	NO	NO	NO
NONE	NO	NO	NO	NO
NONE	NO	NO	NO	NO
NONE	NO	NO	NO	NO
NONE	NO	NO	NO	NO
NONE	NO	NO	NO	NO
NONE	NO	NO	NO	NO
S. aureus	NO	NO	NO	NO
NONE	NO	NO	NO	NO
NONE	NO	NO	NO	NO
NONE	NO	NO	NO	NO
NONE	NO	NO	NO	NO
NONE	NO	NO	NO	NO
NONE	NO	NO	NO	NO
NONE	NO	NO	NO	NO
NONE	NO	NO	NO	NO
NONE	NO	NO	NO	NO
NONE	NO	NO	NO	NO
S. aureus	YES	NO	NO	NO
NONE	NO	NO	NO	NO
S. saprophyticus	NO	NO	NO	NO
NONE	NO	NO	NO	NO
S. aureus	YES	NO	NO	NO
NONE	NO	NO	NO	NO
S. aureus	YES	NO	NO	NO
S. aureus	YES	NO	NO	NO
NONE	NO	NO	NO	NO
NONE	NO	NO	NO	NO
NONE	NO	NO	NO	NO
NONE	NO	NO	NO	NO
S. aureus	YES	NO	NO	NO
S. aureus	YES	NO	NO	NO
S. saprophyticus	YES	NO	NO	NO
NONE	NO	NO	NO	NO
NONE	NO	NO	NO	NO
NONE	NO	NO	NO	NO
NONE	NO	NO	NO	NO
NONE	NO	NO	NO	NO
NONE	NO	NO	NO	NO
NONE	NO	NO	NO	NO
NONE	NO	NO	NO	NO
S. saprophyticus	NO	NO	NO	NO
S. aureus	NO	NO	NO	NO
NONE	NO	NO	NO	NO
NONE	NO	NO	NO	NO
NONE	NO	NO	NO	NO
NONE	NO	NO	NO	NO
S. aureus	YES	NO	NO	NO
S. saprophyticus	NO	NO	NO	NO
NONE	NO	NO	NO	NO
NONE	NO	NO	NO	NO
S. aureus	YES	NO	NO	NO
NONE	NO	NO	NO	NO
NONE	NO	NO	NO	NO
NONE	NO	NO	NO	NO
S. saprophyticus	NO	NO	YES	YES
NONE	NO	NO	NO	NO
NONE	NO	NO	NO	NO
S. saprophyticus	NO	NO	NO	NO
NONE	NO	NO	NO	NO
NONE	NO	NO	NO	NO
NONE	NO	NO	NO	NO
NONE	NO	NO	NO	NO
S. aureus	YES	NO	NO	NO
S. warneri	NO	NO	NO	NO
NONE	NO	NO	NO	NO
S. aureus	YES	NO	NO	NO
S. saprophyticus	NO	NO	NO	NO
S. saprophyticus	YES	NO	NO	NO
S. aureus	YES	NO	NO	NO
NONE	NO	NO	NO	NO
NONE	NO	NO	NO	NO
S. saprophyticus	YES	NO	NO	NO
NONE	NO	NO	NO	NO
S. saprophyticus	NO	NO	YES	NO
NONE	NO	NO	NO	NO
NONE	NO	NO	NO	NO
NONE	NO	NO	NO	NO
NONE	NO	NO	NO	NO
NONE	NO	NO	NO	NO
S. aureus	NO	NO	NO	NO
S. saprophyticus	NO	NO	NO	NO
NONE	NO	NO	NO	NO
NONE	NO	NO	NO	NO
NONE	NO	NO	NO	NO
NONE	NO	NO	NO	NO
S. saprophyticus	NO	NO	NO	NO
NONE	NO	NO	NO	NO
NONE	NO	NO	NO	NO
NONE	NO	NO	NO	NO
S. aureus	YES	NO	NO	NO
NONE	NO	NO	NO	NO
NONE	NO	NO	NO	NO
S. aureus	YES	NO	NO	NO
NONE	NO	NO	NO	NO
S. aureus	NO	NO	NO	NO
S. aureus	YES	YES	NO	YES
NONE	NO	NO	NO	NO
NONE	NO	NO	NO	NO
NONE	NO	NO	NO	NO
NONE	NO	NO	NO	NO
S. aureus	YES	NO	NO	NO
S. saprophyticus	NO	NO	NO	NO
NONE	NO	NO	NO	NO
NONE	NO	NO	NO	NO
S. warneri	NO	NO	NO	NO
NONE	NO	NO	NO	NO
NONE	NO	NO	NO	NO
S. warneri	NO	NO	NO	NO
NONE	NO	NO	NO	NO
NONE	NO	NO	NO	NO
NONE	NO	NO	NO	NO
NONE	NO	NO	NO	NO
NONE	NO	NO	NO	NO
S. aureus	YES	NO	NO	NO
S. aureus	NO	NO	NO	NO
NONE	NO	NO	NO	NO
S. saprophyticus	YES	NO	NO	NO
NONE	NO	NO	NO	NO

NONE	NO	NO	NO	NO
NONE	NO	NO	NO	NO
NONE	NO	NO	NO	NO
NONE	NO	NO	NO	NO
S. aureus	YES	NO	NO	NO
NONE	NO	NO	NO	NO
NONE	NO	NO	NO	NO
S. xylosus	YES	NO	NO	NO
S. aureus	NO	NO	NO	NO
S. saprophyticus	NO	NO	NO	NO
S. aureus	YES	NO	NO	NO
NONE	NO	NO	NO	NO
S. aureus	YES	NO	NO	NO
S. aureus	YES	NO	NO	NO
NONE	NO	NO	NO	NO
NONE	NO	NO	NO	NO
S. aureus	YES	NO	NO	NO
NONE	NO	NO	NO	NO
NONE	NO	NO	NO	NO
S. aureus	YES	NO	NO	NO
NONE	NO	NO	NO	NO
NONE	NO	NO	NO	NO
NONE	NO	NO	NO	NO
S. saprophyticus	NO	NO	NO	NO
NONE	NO	NO	NO	NO
NONE	NO	NO	NO	NO
NONE	NO	NO	NO	NO
NONE	NO	NO	NO	NO
NONE	NO	NO	NO	NO
NONE	NO	NO	NO	NO
NONE	NO	NO	NO	NO
NONE	NO	NO	NO	NO
NONE	NO	NO	NO	NO
NONE	NO	NO	NO	NO
NONE	NO	NO	NO	NO
NONE	NO	NO	NO	NO
NONE	NO	NO	NO	NO
NONE	NO	NO	NO	NO
S. aureus	NO	NO	NO	NO
NONE	NO	NO	NO	NO
NONE	NO	NO	NO	NO
NONE	NO	NO	NO	NO
S. aureus	YES	NO	NO	NO
NONE	NO	NO	NO	NO
S. warneri	YES	NO	NO	NO
NONE	NO	NO	NO	NO
NONE	NO	NO	NO	NO
NONE	NO	NO	NO	NO
NONE	NO	NO	NO	NO
S. aureus	YES	NO	NO	NO
NONE	NO	NO	NO	NO
NONE	NO	NO	NO	NO
NONE	NO	NO	NO	NO
S. aureus	YES	NO	NO	NO
NONE	NO	NO	NO	NO
NONE	NO	NO	NO	NO
NONE	NO	NO	NO	NO
NONE	NO	NO	NO	NO
NONE	NO	NO	NO	NO
NONE	NO	NO	NO	NO
NONE	NO	NO	NO	NO
NONE	NO	NO	NO	NO
NONE	NO	NO	NO	NO
S. aureus	YES	NO	NO	NO
NONE	NO	NO	NO	NO
NONE	NO	NO	NO	NO
NONE	NO	NO	NO	NO
S. aureus	NO	NO	NO	NO
NONE	NO	NO	NO	NO
S. aureus	YES	NO	NO	NO
S. aureus	NO	NO	NO	NO
NONE	NO	NO	NO	NO
NONE	NO	NO	NO	NO
S. saprophyticus	YES	NO	NO	NO
S. aureus	YES	NO	NO	NO
NONE	NO	NO	NO	NO
NONE	NO	NO	NO	NO
NONE	NO	NO	NO	NO
NONE	NO	NO	NO	NO
NONE	NO	NO	NO	NO
NONE	NO	NO	NO	NO
NONE	NO	NO	NO	NO
NONE	NO	NO	NO	NO
S. xylosus	YES	NO	NO	NO
NONE	NO	NO	NO	NO
NONE	NO	NO	NO	NO
NONE	NO	NO	NO	NO
S. aureus	NO	NO	NO	NO
S. saprophyticus	NO	YES	NO	NO
S. aureus	YES	NO	NO	NO
NONE	NO	NO	NO	NO
S. aureus	YES	NO	NO	NO
S. aureus	YES	NO	NO	NO
S. aureus	YES	NO	NO	NO
S. aureus	YES	NO	NO	NO
S. aureus	NO	NO	NO	NO
S. aureus	NO	NO	NO	NO
NONE	NO	NO	NO	NO
NONE	NO	NO	NO	NO
S. aureus	YES	NO	NO	NO
NONE	NO	NO	NO	NO
NONE	NO	NO	NO	NO
NONE	NO	NO	NO	NO
S. aureus	YES	NO	NO	NO
NONE	NO	NO	NO	NO
S. aureus	YES	NO	NO	NO
NONE	NO	NO	NO	NO
NONE	NO	NO	NO	NO
NONE	NO	NO	NO	NO
NONE	NO	NO	NO	NO
NONE	NO	NO	NO	NO
S. aureus	YES	NO	NO	NO
NONE	NO	NO	NO	NO
NONE	NO	NO	NO	NO
NONE	NO	NO	NO	NO
NONE	NO	NO	NO	NO
S. aureus	YES	NO	NO	NO
S. aureus	NO	NO	NO	NO
S. aureus	YES	NO	NO	NO
NONE	NO	NO	NO	NO
NONE	NO	NO	NO	NO
S. aureus	NO	NO	NO	NO
NONE	NO	NO	NO	NO
NONE	NO	NO	NO	NO
NONE	NO	NO	NO	NO
NONE	NO	NO	NO	NO
NONE	NO	NO	NO	NO
NONE	NO	NO	NO	NO
S. aureus	YES	NO	NO	NO

S. haemolyticus	YES	NO	NO	NO
NONE	NO	NO	NO	NO
S. aureus	YES	NO	NO	YES
NONE	NO	NO	NO	NO
NONE	NO	NO	NO	NO
NONE	NO	NO	NO	NO
S. aureus	NO	NO	NO	NO
NONE	NO	NO	NO	NO
S. aureus	NO	NO	NO	NO
NONE	NO	NO	NO	NO
NONE	NO	NO	NO	NO
NONE	NO	NO	NO	NO
NONE	NO	NO	NO	NO
NONE	NO	NO	NO	NO
NONE	NO	NO	NO	NO
S. aureus	YES	NO	NO	NO
NONE	NO	NO	NO	NO
NONE	NO	NO	NO	NO
NONE	NO	NO	NO	NO
S. warneri	YES	NO	NO	NO
NONE	NO	NO	NO	NO
NONE	NO	NO	NO	NO
NONE	NO	NO	NO	NO
NONE	NO	NO	NO	NO
S. aureus	YES	NO	NO	NO
NONE	NO	NO	NO	NO
S. aureus	YES	NO	NO	NO
NONE	NO	NO	NO	NO
NONE	NO	NO	NO	NO
NONE	NO	NO	NO	NO
NONE	NO	NO	NO	NO
NONE	NO	NO	NO	NO
NONE	NO	NO	NO	NO
NONE	NO	NO	NO	NO
S. aureus	YES	NO	NO	NO
S. aureus	NO	NO	NO	NO
S. aureus	NO	NO	NO	NO
NONE	NO	NO	NO	NO
NONE	NO	NO	NO	NO
NONE	NO	NO	NO	NO
S. aureus	YES	NO	NO	NO
NONE	NO	NO	NO	NO
NONE	NO	NO	NO	NO
NONE	NO	NO	NO	NO
S. aureus	YES	NO	NO	NO
NONE	NO	NO	NO	NO
NONE	NO	NO	NO	NO
NONE	NO	NO	NO	NO
NONE	NO	NO	NO	NO
NONE	NO	NO	NO	NO
NONE	NO	NO	NO	NO
NONE	NO	NO	NO	NO
NONE	NO	NO	NO	NO
S. aureus	YES	YES	NO	NO
NONE	NO	NO	NO	NO
NONE	NO	NO	NO	NO
NONE	NO	NO	NO	NO
S. cohnii	YES	NO	NO	NO
NONE	NO	NO	NO	NO
NONE	NO	NO	NO	NO
S. aureus	YES	NO	NO	NO
NONE	NO	NO	NO	NO
NONE	NO	NO	NO	NO
NONE	NO	NO	NO	NO
NONE	NO	NO	NO	NO
NONE	NO	NO	NO	NO
NONE	NO	NO	NO	NO
NONE	NO	NO	NO	NO
NONE	NO	NO	NO	NO
NONE	NO	NO	NO	NO
NONE	NO	NO	NO	NO
NONE	NO	NO	NO	NO
NONE	NO	NO	NO	NO
NONE	NO	NO	NO	NO
NONE	NO	NO	NO	NO
NONE	NO	NO	NO	NO
NONE	NO	NO	NO	NO
NONE	NO	NO	NO	NO
NONE	NO	NO	NO	NO
NONE	NO	NO	NO	NO
NONE	NO	NO	NO	NO
NONE	NO	NO	NO	NO
NONE	NO	NO	NO	NO
NONE	NO	NO	NO	NO
NONE	NO	NO	NO	NO
NONE	NO	NO	NO	NO
NONE	NO	NO	NO	NO
NONE	NO	NO	NO	NO
NONE	NO	NO	NO	NO
S. aureus	NO	NO	NO	NO
S. aureus	YES	NO	NO	NO
S. aureus	NO	NO	NO	NO
S. aureus	YES	NO	NO	NO
NONE	NO	NO	NO	NO
NONE	NO	NO	NO	NO
NONE	NO	NO	NO	NO
NONE	NO	NO	NO	NO
NONE	NO	NO	NO	NO
NONE	NO	NO	NO	NO
NONE	NO	NO	NO	NO
NONE	NO	NO	NO	NO
NONE	NO	NO	NO	NO
NONE	NO	NO	NO	NO
NONE	NO	NO	NO	NO
S. aureus	YES	NO	NO	NO
NONE	NO	NO	NO	NO
NONE	NO	NO	NO	NO
S. aureus	YES	NO	NO	NO
NONE	NO	NO	NO	NO
S. aureus	NO	NO	NO	NO
S. aureus	NO	NO	NO	NO
NONE	NO	NO	NO	NO
S. aureus	YES	NO	NO	NO
NONE	NO	NO	NO	NO
S. aureus	YES	NO	NO	NO
NONE	NO	NO	NO	NO
NONE	NO	NO	NO	NO
