# Supplementary material for: Prevalence and antibiotic resistance of Staphylococcus aureus associated with a college-aged cohort: life-style factors that contribute to nasal carriage
Source: Front Cell Infect Microbiol. 2023 Jun 27;13:1195758. doi: 10.3389/fcimb.2023.1195758 (PMC10333693; doi:10.3389/fcimb.2023.1195758)
Supplement: Supplementary file 4 [file DataSheet_4.pdf]

```
import numpy as np
import itertools as it
from scipy.stats import fisher_exact
from scipy.stats import spearmanr
import matplotlib.pyplot as plt

path = '/media/tbecker/data/staph_view/staph_project_v1.4.tsv'
factors =
['Staph_Exposure','Healthcare_Exposure','Antibiotics_Exposure','Pet_Ex
posure',

'Tobacco_Usage','Piercings_and_Tats','Public_Transport_Usage','Shave_o
r_Makeup',

'Weekday_Sleep','Weekend_Sleep_Offset','Housing','Sheet_Change','Bathr
oom_Mates',

'Gym_Time','Sports_and_Equipment','Musical_Instrument_Usage']
with open(path,'r') as f:
    raw = [line.replace('\n','').rsplit('\t') for line in
f.readlines()]
    header,data = raw[0],raw[1:]
    c_idx = {header[i]:i for i in range(len(header))}
    for i in range(len(data)): #
transformations-------------------------------------------------------
------------------
        data[i][c_idx['Sports_and_Equipment']]     = ('YES' if data[i]
[c_idx['Sports_and_Equipment']]!='NO' else 'NO')
        data[i][c_idx['Staph_Exposure']]           = ('YES' if data[i]
[c_idx['Staph_Exposure']]!='NO' else 'NO')
        data[i][c_idx['Musical_Instrument_Usage']] = ('YES' if data[i]
[c_idx['Musical_Instrument_Usage']]!='NO' else 'NO')
        data[i][c_idx['Housing']] = ('YES' if data[i]
[c_idx['Housing']]=='Dorm' or data[i][c_idx['Housing']]=='On-Campus'
else 'NO')
        data[i][c_idx['Gym_Time']] = ('YES' if data[i]
[c_idx['Gym_Time']]!='15' else 'NO')
        data[i][c_idx['Sheet_Change']] = ('YES' if int(data[i]
[c_idx['Sheet_Change']])>2 else 'NO')
        data[i][c_idx['Tobacco_Usage']] = ('YES' if float(data[i]
[c_idx['Tobacco_Usage']])>0 else 'NO')
        data[i][c_idx['Weekday_Sleep']] = ('YES' if float(data[i]
[c_idx['Weekday_Sleep']])>7 else 'NO')
        #Watson NF, Badr MS, Belenky G, et al. Recommended amount of
sleep for a healthy adult: a joint consensus statement of the American
Academy of Sleep Medicine and Sleep Research Society. Sleep.
2015;38(6):843–844.
        data[i][c_idx['Weekend_Sleep_Offset']] = ('YES' if
float(data[i][c_idx['Weekend_Sleep_Offset']])>1 else 'NO')
        data[i][c_idx['Healthcare_Exposure']] = ('YES' if
```

```
float(data[i][c_idx['Healthcare_Exposure']])>0 else 'NO')
        data[i][c_idx['Antibiotics_Exposure']] = ('YES' if
float(data[i][c_idx['Antibiotics_Exposure']])>0 else 'NO')
        data[i][c_idx['Bathroom_Mates']] = ('YES' if float(data[i]
[c_idx['Bathroom_Mates']])>0 else 'NO')
        data[i][c_idx['Piercings_and_Tats']] = ('YES' if float(data[i]
[c_idx['Piercings_and_Tats']])>0 else 'NO')
        data[i][c_idx['Shave_or_Makeup']] = ('YES' if float(data[i]
[c_idx['Shave_or_Makeup']])>0 else 'NO')
        data[i][c_idx['Public_Transport_Usage']] = ('YES' if
float(data[i][c_idx['Public_Transport_Usage']])>0 else 'NO')
        data[i][c_idx['Pet_Exposure']] = ('YES' if float(data[i]
[c_idx['Pet_Exposure']])>0 else 'NO')
species = sorted(set([row[-5] for row in data]))
species = species[1:] #take out NONE

#predictive model limits?
from sklearn.linear_model import LogisticRegression
from sklearn.neural_network import MLPClassifier

def f1_accuracy(T,P):
    a,b,n,m = 0,0,0,0
    for i in range(len(T)):
        if T[i]==0:
            if P[i]==0: a += 1
            else:       m += 1
        else:
            n += 1
            if P[i]==1:
                b += 1
                m += 1
    prec,rec,f1 = 0.0,0.0,0.0
    if m>0.0:        prec = b/m
    if n>0.0:        rec  = a/n
    if prec+rec>0.0: f1 = 2.0*(prec*rec)/(prec+rec)
    return f1

# F = []#{factor:[] for factor in factors}
# for i in range(1):
#     if i>0 and i%100==0:
print('i=%s----------------------------------------'%i)
#     # for factor in factors:
#     x_data,y_data = [],[]
#     for i in range(len(data)):
#         x_data += [[1 if data[i][c_idx[v]]=='YES' else 0 for v in
factors]]
#         y_data += [1 if data[i][c_idx['Species']]=='S. aureus' else
0]
#     x_data = np.asarray(x_data,dtype=float)
#     y_data = np.asarray(y_data)
```

```
#     train_idx =
sorted(np.random.choice(range(len(x_data)),int(round(len(x_data)*0.7))
,replace=False))
#     test_idx  =
sorted(set(range(len(x_data))).difference(set(train_idx)))
#
#     ner  = MLPClassifier(alpha=1e-5,hidden_layer_sizes=(4,
2),max_iter=1000).fit(x_data[train_idx],y_data[train_idx])
#     model_pred = ner.predict(x_data[test_idx])
#     obs_prop   = 1.0-sum(y_data[train_idx])/len(train_idx)
#overestimate since we will do f1...
#     m_acc  = f1_accuracy(y_data[test_idx],model_pred)
#
#     F += [m_acc-obs_prop]
# for factor in factors:
#     print('factor=%s had %s higher predictive power on %s'%
(factor,round(np.mean(F),2),species[0]))

for spec in species[0:1]:
    staph_counts = {'+':[],'-':[]}
    for i in range(len(data)):
        if data[i][c_idx['Gender']] != 'X': #exclusion
logic------------------------
            if data[i][c_idx['Species']]==spec: staph_counts['+'] +=
[[data[i][c_idx[v]] for v in factors]]
            else:                              staph_counts['-'] +=
[[data[i][c_idx[v]] for v in factors]]

    i_idx = {i:factors[i] for i in range(len(factors))}
    H = {}
    for s in staph_counts:
        H[s] = {factors[i]:{'YES':0,'NO':0} for i in
range(len(factors))}
        for row in staph_counts[s]:
            for i in range(len(row)):
                v = i_idx[i]
                if row[i] in H[s][v]: H[s][v][row[i]] += 1
                else:                 H[s][v][row[i]]  = 1

    H['+rate'] = {}
    for v in factors:
        H['+rate'][v] = {}
        for l in H['+'][v]:
            H['+rate'][v][l] = round(H['+'][v][l]/(H['+'][v][l]+H['-']
[v][l]),2)

    H['+p'] = {}
    for v in factors:
        H['+p'][v] = {}
        v_sum = sum([H['+'][v][l] for l in H['+'][v]])
```

```
        H['+p'][v] = {l:round((H['+'][v][l]/v_sum if v_sum>0 else
0.0),2) for l in H['+'][v]}

    H['-rate'] = {}
    for v in factors:
        H['-rate'][v] = {}
        for l in H['-'][v]:
            H['-rate'][v][l] = round(H['-'][v][l]/(H['+'][v][l]+H['-']
[v][l]),2)

    H['-p'] = {}
    for v in factors:
        H['-p'][v] = {}
        v_sum = sum([H['-'][v][l] for l in H['-'][v]])
        H['-p'][v] = {l:round((H['-'][v][l]/v_sum if v_sum>0 else
0.0),2) for l in H['-'][v]}

    #build 2x2 fisher contingency tables
    T,F = {},{}
    for v in factors:
        T[v] = np.array([[H['+'][v][l] for l in ['YES','NO']],[H['-']
[v][l] for l in ['YES','NO']]])
        ods,F[v] = fisher_exact(T[v],alternative='greater')
        # print('%s: %s'%(v,round(F[v],3)))

    p_value = 0.15
    sigs = []
    for v in factors:
        if F[v]<=p_value: sigs += [[v,F[v]]]
    if len(sigs)>0:
        print('\nSpecies="%s" Fisher Exact Test: p_value of random
sample > than observed factor:'%spec)
        for sig in sigs:
            print('\t%s: %s'%(sig[0].ljust(25),round(sig[1],3)))

    #build a numeric matrix that has staph_areus as row 0...
    staph = [(1 if data[i][c_idx['Species']]==spec else 0) for i in
range(len(data))]
    M = []
    for v in factors:
        row = [(1 if data[i][c_idx[v]]=='YES' else 0)for i in
range(len(data))]
        M += [row]
    M = np.array(M)
    n = M.shape[0]
    S = np.zeros((n+1,n+1),dtype=float)
    C = {}
    for i in range(n):
        corr = spearmanr(staph,M[i])
        if corr[1]<=p_value: C[('Staph',factors[i])] = [round(x,2) for
```

```
x in corr]

    for i,j in sorted(it.combinations(range(n),2)):
        corr = spearmanr(M[i],M[j])
        S[j,i] = S[i,j] = round(corr[0],2)
        if corr[1]<=p_value: C[(factors[i],factors[j])] = [round(x,2)
for x in corr]
    # print('\nSpecies="%s" Spearman Correlation Coefficent and
p_value of random sample having greater value:'%spec)
    # for c in C:
    #     if c[0]=='Staph' or c[1]=='Staph': print('%s: %s'%
(c[1],C[c]))

    #lets look at positive with respect to resistance...
    res = {k:{'+':0,'-':0} for k in header[-4:]}
    res_pos = False
    for row in data:
        if row[c_idx['Species']]==spec:
            for k in res:
                if row[c_idx[k]]=='YES':
                    res[k]['+']+=1
                    res_pos = True
                else:
                    res[k]['-']+=1
    if len(sigs)>0 and res_pos:
        print('Species="%s" +Resistance:'%spec)
        for k in res:
            if res[k]['+']>0:
                res_prop = round(res[k]['+']/(res[k]['+']+res[k]
['-']),2)
                print('\t%s: %s'%(k.ljust(25),res_prop)+r'%')
```
