# Supplementary material for: Prevalence and antibiotic resistance of Staphylococcus aureus associated with a college-aged cohort: life-style factors that contribute to nasal carriage
Source: Front Cell Infect Microbiol. 2023 Jun 27;13:1195758. doi: 10.3389/fcimb.2023.1195758 (PMC10333693; doi:10.3389/fcimb.2023.1195758)
Supplement: Supplementary file 5 [file Table_1.docx]

**TABLE S1** PCR Primers, Annealing Temperatures, and Amplicon Sizes

| **Gene** | **Name** | **Primer sequence (5’ – 3’)** | **Annealing Temperature** | **Amplicon Size** | **Reference** |
| --- | --- | --- | --- | --- | --- |
|  |  |  |  |  |  |
| *16S rRNA* | 27F | AGAGTTTGATCMTGGCTCAG | 51 | 1466 | Lane, 1991 |
|  | 1492R | GGTTACCTTGTTACGACTT |  |  | Turner et al., 1999 |
|  |  |  |  |  |  |
| *blaZ* | blaZ-1 | AAGAGATTTGCCTATGCTTC | 55 | 517 | Gao et al., 2011 |
|  | blaZ-2 | GCTTGACCACTTTTATCAGC |  |  |  |
|  |  |  |  |  |  |
| *mecA* | mecA-1 | AAAATCGATGGTAAAGGTTGGC | 55.5 | 533 | Murakami et al., 1991 |
|  | mecA-2 | AGTTCTGCAGTACCGGATTTGC |  |  |  |
|  |  |  |  |  |  |
| *tetK* | tetK-1 | TCGATAGGAACAGCAGTA | 55 | 169 | Gao et al., 2011 |
|  | tetK-2 | CAGCAGATCCTACTCCTT |  |  |  |
|  |  |  |  |  |  |
| *tetM* | tetM-1 | CCGCACCCTCTACTACAA | 56 | 351 | Gao et al., 2011 |
|  | tetM-2 | CATTCCACTTCCCAACG |  |  |  |
|  |  |  |  |  |  |
| *tetO* | tetO-F | AACTTAGGCATTCTGGCTCAC | 55 | 515 | Ng et al., 2001 |
|  | tetO-R | TCCCACTGT TCCATATCGTCA |  |  |  |
|  |  |  |  |  |  |
| *tetL* | tetL-F | TCGTTAGCGTGCTGTCATTC | 55 | 267 | Ng et al., 2001 |
|  | tetL-R | GTATCCCACCAATGTAGCCG |  |  |  |
|  |  |  |  |  |  |
| *dfrS1* | dfrS1-1 | CACTTGTAATGGCACGGAAA | 48 | 270 | Argudín et al., 2011 |
|  | dfrS1-2 | CGAATGTGTATGGTGGAAAG |  |  |  |
|  |  |  |  |  |  |
| *pbp3* | pbp3-G88A-F | TCAAATGATGAAATCGTTCAAAA | 54 | 277 | Chadwick et al., 2013 |
|  | pbp3-G88A-R | TCCGATTGTGTTGTTTTTCG |  |  |  |
|  |  |  |  |  |  |
| *norA* | NorA-Fw | TTCACCAAGCCATCAAAAAG | 45 | 620 | Couto et al., 2008 |
|  | NorA-Rv | CTTGCCTTTCTCCAGCAATA |  |  |  |
|  |  |  |  |  |  |
| *ermA* | ermA-F | GTTCAAGAACAATCAATACAGAG | 52 | 421 | Lina et al., 1999 |
|  | ermA-R | GGATCAGGAAAAGGACATTTTAC |  |  |  |
|  |  |  |  |  |  |
| *ermC* | ermC-F | GCTAATATTGTTTAAATCGTCAATTCC | 52 | 572 | Lina et al., 1999 |
|  | ermC-R | GGATCAGGAAAAGGACATTTTAC |  |  |  |
|  |  |  |  |  |  |
| *msrA* | msrA-F | GGCACAATAAGAGTGTTTAAAGG | 50 | 940 | Lina et al., 1999 |
|  | msrA-R | AAGTTATATCATGAATAGATTGTCCTGTT |  |  |  |
|  |  |  |  |  |  |

Argudín, M. A., Tenhagen, B. A., Fetsch, A., Sachsenröder, J., Käsbohrer, A., Schroeter, A., et al. (2011). Virulence and resistance determinants of German Staphylococcus aureus ST398 isolates from nonhuman sources. *Appl Environ Microbiol* 77, 3052–3060. doi: 10.1128/AEM.02260-10.

Chadwick, S. G., Prasad, A., Smith, W. L., Mordechai, E., Adelson, M. E., and Gygax, S. E. (2013). Detection of epidemic USA300 community-associated methicillin-resistant Staphylococcus aureus strains by use of a single allele-specific PCR assay targeting a novel polymorphism of Staphylococcus aureus pbp3. *J Clin Microbiol* 51, 2541–2550. doi: 10.1128/JCM.00417-13.

Couto, I., Costa, S. S., Viveiros, M., Martins, M., and Amaral, L. (2008). Efflux-mediated response of Staphylococcus aureus exposed to ethidium bromide. *Journal of Antimicrobial Chemotherapy* 62, 504–513. doi: 10.1093/jac/dkn217.

Gao, J., Ferreri, M., Liu, X. Q., Chen, L. B., Su, J. L., and Han, B. (2011). Development of multiplex polymerase chain reaction assay for rapid detection of Staphylococcus aureus and selected antibiotic resistance genes in bovine mastitic milk samples. *Journal of Veterinary Diagnostic Investigation* 23, 894–901. doi: 10.1177/1040638711416964.

Lina, G., Quaglia, A., Reverdy, M.-E., Leclercq, R., Vandenesch, F. O., and Etienne, J. (1999). Distribution of Genes Encoding Resistance to Macrolides, Lincosamides, and Streptogramins among Staphylococci.

Murakami, K., Minamide, W., Wada, K., Nakamura,’, E., Teraoka, H., and Watanabe’, S. (1991). Identification of Methicillin-Resistant Strains of Staphylococci by Polymerase Chain Reaction.

Ng, L. K., Martin, I., Alfa, M., and Mulvey, M. (2001). Multiplex PCR for the detection of tetracycline resistant genes. *Mol Cell Probes* 15, 209–215. doi: 10.1006/mcpr.2001.0363.

Turner, S., PRYERb, K. M., W M I A, V. P., and PALMERa, J. D. (1999). Investigating Deep Phylogenetic Relationships among Cyanobacteria and Plastids by Small Subunit rRNA Sequence Analysis1.
